# The revealing of a novel lipid transfer protein lineage in green algae

**DOI:** 10.1186/s12870-023-04040-1

**Published:** 2023-01-11

**Authors:** Ming-Der Huang, Chin-Wei Wu, Hong-Yun Chou, Sou-Yu Cheng, Hsin-Yang Chang

**Affiliations:** 1 Department of Biological Sciences, National Sun Yat-sen University, Kaohsiung, Taiwan 80424; 2 Department of Marine Biotechnology and Resources, National Sun Yat-sen University, Kaohsiung, Taiwan 80424; 3 Department of Life Sciences and Institute of Genome Sciences, National Yang Ming Chiao Tung University, Taipei, Taiwan 11221

**Keywords:** Non-specific lipid transfer protein, Green algae, Chlamydomonas, Zygotes

## Abstract

**Background:**

Non-specific lipid transfer proteins (nsLTPs) are a group of small and basic proteins that can bind and transfer various lipid molecules to the apoplastic space. A typical nsLTP carries a conserved architecture termed eight-cysteine motif (8CM), a scaffold of loop-linked helices folding into a hydrophobic cavity for lipids binding. Encoded by a multigene family, nsLTPs are widely distributed in terrestrial plants from bryophytes to angiosperms with dozens of gene members in a single species. Although the nsLTPs in the most primitive plants such as Marchantia already reach 14 members and are divergent enough to form separate groups, so far none have been identified in any species of green algae.

**Results:**

By using a refined searching strategy, we identified putative nsLTP genes in more than ten species of green algae as one or two genes per haploid genome but not in red and brown algae. The analyses show that the algal nsLTPs carry unique characteristics, including the extended 8CM spacing, larger molecular mass, lower p*I* value and multiple introns in a gene, which suggests that they could be a novel nsLTP lineage. Moreover, the results of further investigation on the two Chlamydomonas nsLTPs using transcript and protein assays demonstrated their late zygotic stage expression patterns and the canonical nsLTP properties were also verified, such as the fatty acids binding and proteinase resistance activities.

**Conclusions:**

In conclusion, a novel nsLTP lineage is identified in green algae, which carries some unique sequences and molecular features that are distinguishable from those in land plants. Combined with the results of further examinations of the Chlamydomonas nsLTPs in vitro, possible roles of the algal nsLTPs are also suggested. This study not only reveals the existence of the nsLTPs in green algae but also contributes to facilitating future studies on this enigmatic protein family.

**Supplementary Information:**

The online version contains supplementary material available at 10.1186/s12870-023-04040-1.

## Background

Non-specific lipid transfer proteins (nsLTPs) are a group of small secretory proteins only found in land plants; these proteins are believed to reversibly bind and transfer a wide variety of lipid molecules to the extracellular space [1–3]. With the N-terminal secretory signal peptide (SPs) at the N-terminal end removed, a mature nsLTP, in general, carries a molecular mass in the range of 6 to 10 kDa and a isoelectric point (p*I*) in the range of 8 to 12 [1, 4]. The central region of the nsLTP is composed of an eight-cysteine motif (8CM) with amino acid residues arranged as C-Xn-C-Xn-CC-Xn-CXC-Xn-C-Xn-C, X denotes any amino acid residue, and n is the number of X [5]; this cysteine-rich motif packs into an architecture of four to five loop-linked helices folded into a hydrophobic cavity/tunnel for cargo binding [6, 7]. The four disulfide bonds that are formed by the Cys residues in 8CM maintain the conformational stability of nsLTPs structure and thus makes the proteins resist high temperature and protease activities [8–11]. This unique 8CM pattern is displayed in several protein families of the prolamin superfamily from seed plants, which have different functions and little sequence similarity except for sharing conserved structural features of disulfide bonds [12, 13].

The term “non-specific” of plant nsLTPs came from their broad binding targets ranging from short-chain fatty acids to complex lipids, including saturated and unsaturated fatty acids, hydroxyl fatty acids, phospholipids, cuticular components, and sterol compounds, which bind in several orientations [14–16]. Based on previous reports, most nsLTPs found in land plants, including moss (*Physcomitrella patens*), gymnosperms (*Ginkgo biloba*) and angiosperms (*Nicotiana tabacum* and *Triticum aestivum*) preferred ligands with medium aliphatic chain lengths (C14 to C18) and/or *cis* unsaturated hydrocarbon chains [17–19]. Although the mechanism of how nsLTPs transport lipids is still unclear, the accumulating studies of the expression patterns of nsLTP in different developmental stages and their responses to different biotic and abiotic stresses may improve our knowledge of nsLTP roles [20–22]. Previous studies indicate that nsLTPs could transport the lipid-derived monomers of the barrier materials, such as cutin and waxes, across cell walls to the exterior layers of plants. They either render functions in maintaining the adhesion ability between the hydrophobic cuticle layer and the underlining hydrophilic cell wall or contribute to constitute the barrier tissues, such as the suberin of crown gall periderm and the cross-linked sporopollenin of the pollen wall exterior layer, by providing the aliphatic ingredients of the precursor monomers [23–26]. Furthermore, the property that nsLTPs carry the capabilities to resist pathogens and protease activities has been revealed. For example, some nsLTPs, such as DIR1 (Defective in induced resistance 1) and AZI1 (Azelaic acid-induced 1) in Arabidopsis, play crucial roles in systemic acquired resistance (SAR) toward pathogens in vivo and some nsLTPs are capable of inhibiting fungal growth or show resistance to protease activity in vitro [19, 27–30].

Genes that encode nsLTPs belong to a multigene family with up to 85 members reported in a single species (*Brassia napa*) and are widely distributed in the plant kingdom from basal bryophytes to advanced angiosperms [3, 18, 31]. Plant nsLTPs identified thus far were categorized with several classification systems, but in general, were divided into ten types based on the protein sequence similarity, the interval spacing between each Cys residue in the 8CM, the number and position of introns, and the existence of the Glycophosphatidylinositol (GPI)-anchor modification site at the C-terminus [3, 4, 18, 32]. From the classification system proposed by Edstam et al. (2011), there are five primary types—I, II, C, D, and G, and five minor types that only appear in some specific species—E, F, H, J, and K [32]. At first, the nsLTPs identified in seed plants were classified into type I and type II according to the theoretical molecular mass of the mature proteins. More specifically, a mature protein sequence of type I nsLTPs and type II nsLTPs is composed of approximately 90 amino acids with a molecular mass of 9 kDa and 70 amino acids with a molecular mass of 7 kDa, respectively [33]. Moreover, type I nsLTPs are characterized as carrying a long tunnel-like cavity, the disulfide bridges of which are linked as C^1^-**C**^**6**^, **C**^**5**^-C^8^, C^2^-C^3^, and C^4^-C^7^, while type II nsLTPs, on the other hand, possess two adjacent hydrophobic clefts with the four disulfide bonds linked as C^1^-**C**^**5**^, **C**^**6**^-C^8^, C^2^-C^3^, and C^4^-C^7^, instead [2, 34]. Subsequently, type C nsLTPs, with a comparably lower p*I* (4–7), were identified in seed plants. Type C nsLTPs in Arabidopsis are specifically expressed in the anther tapetum, and their encoded proteins have functions related to the assembly of sporopollenin in the exine layer of pollen wall [4, 23]. The nsLTPs that encode proteins with an extra GPI anchoring motif at the C-terminus were classified as type G nsLTPs, and their functions are related to cuticle deposition and cell wall organization [35–37]. Previous research suggested that the earliest established nsLTP type in plant evolution is the type D nsLTPs; they exist in liverworts, mosses, and the species of vascular plants except for ferns and the genes carry one intron located four nucleotides downstream of the last Cys codon of the 8CM [32]. It is worth noting that type G and type D nsLTPs have been retained in the species of bryophytes to angiosperms with a close evolutionary relationship [38]. Most nsLTP genes of these ten types carry only one intron either located at the same position after the codon encoding C^8^, including type I, type C, type D, and type F, or at various positions, including type G, type J, and type K; whereas the members in type II, type E, and type H carry no introns [32]. In short, the number and position of the introns of nsLTP genes are crucial factors for nsLTP classification.

From previous research, nsLTP genes have been found in several species of the plant kingdom, including angiosperms (e.g., 77 nsLTP genes in *Oryza sativa*, 79 in *Arabidopsis thaliana*, and 85 genes in *B. napa*), gymnosperms (e.g., 42 genes in *Pinus taeda*), lycopods (e.g., 43 genes in *Selaginella moellendorffii*), mosses (e.g., 40 genes in *P patens*), and liverworts (e.g., 14 genes in *Marchantia polymorpha*) [18, 32]. Despite being widely distributed in the plant kingdom, nsLTPs have not been found in any algal species. Nevertheless, researchers did not rule out that possibility because the previous reports were mainly based on the unfinished genome sequences and ETS databases with limited information. Moreover, since the nsLTP genes in the earliest diverging land plant, *M. polymorpha*, already reached 14 and can be categorized into at least two types, including type D and type G, we speculated that nsLTPs might have been evolved in more primitive types of organisms. As such, with more and more algal genomes that are completely sequenced being publicly available, the timing was right for us to revisit this topic.

In this research, we hypothesized that nsLTPs should be present in algal genomes and performed a thorough search in the fully completed genome sequences of several algal species that have been published. Several putative nsLTP candidates were identified in both chlorophytes (green algae) and charophytes (advanced green algae), and analyzed for several features in gene sequences and protein properties. Further investigation of the nsLTP identified in *Chlamydomonas reinhardtii* was also conducted.

## Results

### Putative sequences of nsLTP genes were identified in green algae

**Fig. 1:**
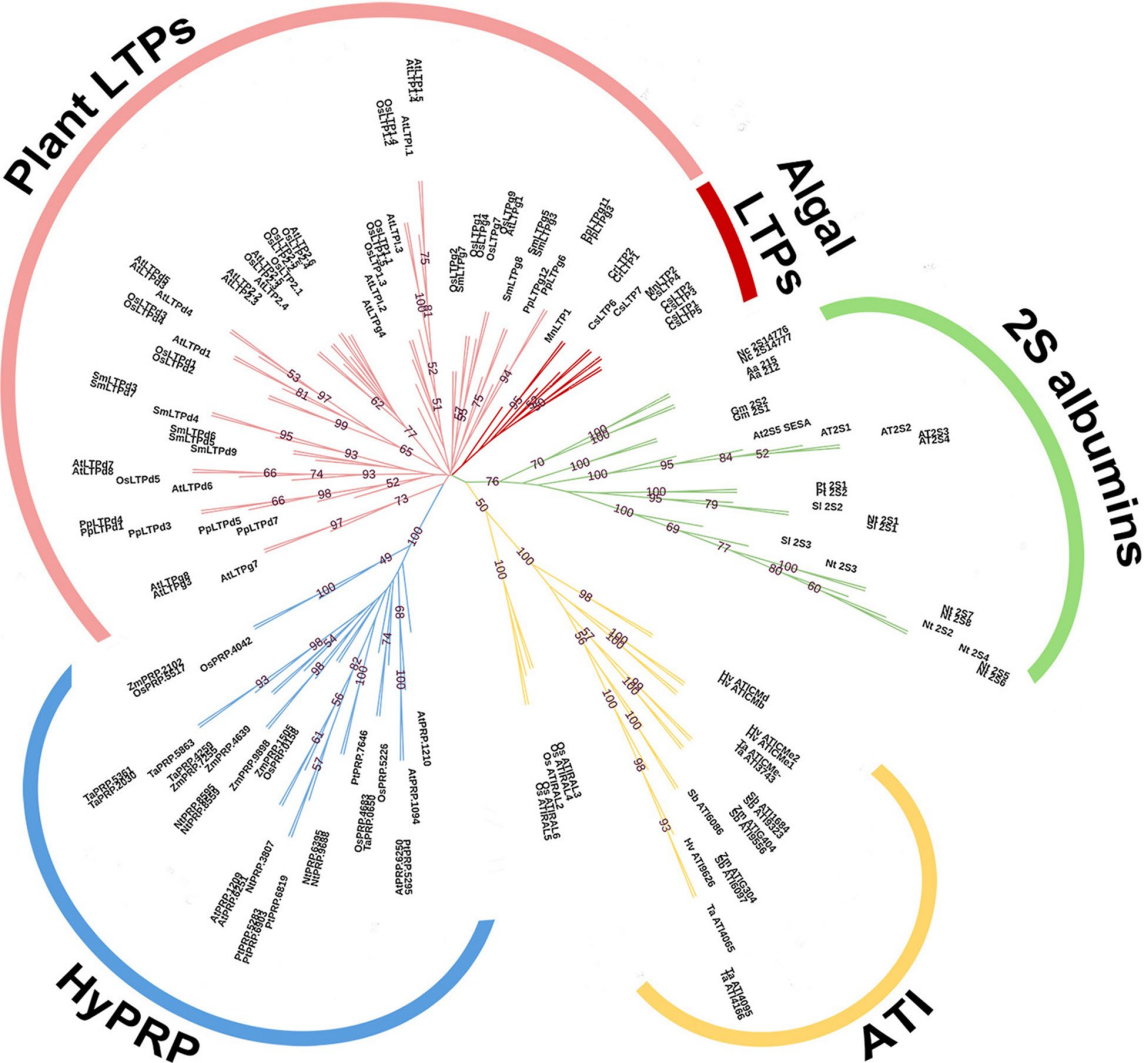
Unrooted phylogenetic tree of the 8CM containing protein families from land plants and green algae. The phylogenetic relationships of the 8CM containing protein in land plants and green algae inferred from the multiple sequence alignments is demonstrated. The tree was constructed by using neighbor-joining method supported by a bootstrap test of 1000 resamplings. The 8CM-containing proteins in plants, including 22 members of amylase trypsin inhibitors (ATIs), 25 members of 2S albumins, 30 hybrid proline-rich proteins (HyPRPs), 61 land plant nsLTPs and 11 members of algal nsLTPs, are depicted in different colors. The number on the node shows the percentage of bootstrap with only those above 50

In an effort to identify the members of nsLTPs in algae, the first attempt we made was to search the algal species that have completely sequenced genomes using the nsLTPs protein sequences of *Arabidopsis thaliana* and *Physcomitrella patens* as baits with BLASTP (the Basic Local Alignment Search Tool: Protein). There were no hits in these algal species despite using a threshold of E-value = 10. This setback could be due to the sequences of algal nsLTPs, if any, being too divergent from those in land plants and thus is difficult to be found by using only land plant nsLTPs as the searching baits in BLAST. Therefore, our attention was shifted to the central protein structure of nsLTPs, that is, the eight-cysteine motif (8CM), which is the hallmark of all land plant nsLTPs. By searching based on the 8CM sequence arrangement, we initially set the number of any amino acid residue X of the C-Xn-C-Xn-CC-Xn-CXC-Xn-C-Xn-C in the same range of plant nsLTPs identified thus far but found nothing in the investigated genomes. Hence, instead of setting the number of X residues from *n* = 8–30 as the canonical land plant nsLTPs, we extended the 8CM spacing to *n* = 8–50 for more flexibility. This modification was made based on the assumption that the basic architecture of nsLTP with extended 8CM spacing sequence is unchanged; that is, still folding into a hydrophobic cavity constituted by four to five α-helices and stabilized by four disulfide bridges. By applying a Perl script with a slight modification to the criterion of the 8CM spacing length, we successfully identified several candidate genes that could encode 8CM-containing proteins in the obtained genome databases of green algae. Subsequently, a further screening process was conducted to eliminate the genes that encode proteins without secretory signal peptides (SPs) or with a molecular mass greater than 60 kDa. After using the strategy to search in the algal genomes of 35 species, we ultimately found 29 nsLTP genes from one charophytes and 12 chlorophytes of green algae (Table 1). When using the online motif-searching tool provided by GenomeNet based on the database of Pfam to search for possible protein motifs that could be encoded by these putative genes, half of the algal nsLTPs were recognized as the LTP2 motif-containing proteins; this analysis somehow provided a positive result for the authenticity of these algal nsLTPs (data not shown). Next, considering that several protein families in the plant kingdom other than nsLTPs also possess both SPs and the 8CM region to which these putative proteins might belong, we conducted a phylogenetic analysis to eliminate this possibility. These other SPs and 8CM-containing protein families include amylase trypsin inhibitors (ATIs), 2S albumins, and hybrid proline-rich proteins (HyPRP) [5]. The representative protein sequences from each of those protein families and the sequences of the algal nsLTPs identified in this research were subjected to multiple sequence alignment using the MAFFT program, followed by phylogenetic analysis with an inferred tree constructed using neighbor-joining methods [39, 40]. In Fig. 1, only the protein sequences of 2S albumins, ATIs and HyPRPs formed separate clades supported with high bootstrap values in the phylogenetic tree. The sequences encoded by the algal nsLTP genes identified in this work formed a mono-clade, located to the same cluster with the representative protein sequences of nsLTPs. These results suggested that the algal nsLTP candidates have a closer phylogenetic relationship with plant nsLTPs than with other 8CM-containing protein families and thus exclude the possibility of the proteins belonging to the protein family other than nsLTPs.

**Table 1:** The number of nsLTPs in the algal species with complete genome sequences

Species	No. of nsLTP	Database
Charophyta		
*Chara braunii*	0	JGI
*Klebsormidium nitens*	2	JGI
Chlorophyta		
*Auxenochlorella protothecoides*	2	NCBI
*Chlamydomonas reinhardtii*	2	JGI
*Chlorella variabilis*	2	NCBI
*Coccomyxa* sp. C-169	8	NCBI
*Gonium pectoral*	1	NCBI
*Micractinium conductrix*	2	NCBI
*Monoraphidium neglectum*	2	NCBI
*Ostreococcus tauri*	2	NCBI
*Ostreobium quekettii*	2	NCBI
*Raphidocelis subcapitata*	2	NCBI
*Tetrabaena socialis*	1	NCBI
*Volvox carteri*	1	JGI
*Astrephomene gubernaculifera*	0	NCBI
*Bathycoccus prasinos*	0	NCBI
*Chlorella* NC64A	0	NCBI
*Chlorokybus atmophyticus*	0	NCBI
*Dunaliella salina*	0	NCBI
*Edaphochlamys debaryana*	0	NCBI
*Haematococcus lacustris*	0	NCBI
*Helicosporidium* sp. ATCC 50920	0	NCBI
*Micromonas pusilla*	0	NCBI
Phaeophyta		
*Cladosiphon okamuranus*	0	JGI
*Ectocarpus siliculosus*	0	NCBI
*Nemacystus decipiens* Onna-1	0	JGI
*Saccharina japonica*	0	NCBI
*Undaria pinnatifida*	0	JGI
Rhodophyta		
*Chondrus crispus*	0	NCBI
*Cyanidiococcus yangmingshanensis*	0	NCBI
*Cyanidioschyzon merolae*	0	NCBI
*Galdieria sulphuraria*	0	NCBI
*Gracilaria domingensis*	0	NCBI
*Porphyra umbilicalis*	0	NCBI
*Porphyridium purpureum*	0	NCBI

The genomes of 35 algal species, including two charophytes and 21 chlorophytes in green algae, five in Phaeophyta, and seven in Rhodophyta, retrieved from NCBI and JGI, and the number of nsLTPs identified in this report are listed

As listed in Table 1, we identified one putative nsLTP gene in each of the following three algal species: *Gonium pectoral*, *Tetrabaena socialis*, *Volvox carteri*, two nsLTP genes in each of the following nine algal species: *Klebsormidium nitens*, *Auxenochlorella protothecoides*, *Chlamydomonas reinhardtii*, *Chlorella variabilis*, *Micractinium conductrix*, *Monoraphidium neglectum*, *Ostreococcus tauri*, *Ostreobium quekettii*, *Raphidocelis subcapitata* and identified eight nsLTPs in *Coccomyxa* sp. C-169 (Table 1). No genes coding for nsLTPs or nsLTP-like proteins were found in the other ten green algae species listed in Table 1, including *Chara braunii*, *Astrephomene gubernaculifera*, *Bathycoccus prasinos*, *Chlorella variabilis* NC64A*, Chlorokybus atmophyticus*, *Dunaliella salina*, *Edaphochlamys debaryana*, *Haematococcus lacustris*, *Haematococcus* sp. ATCC 50920, and *Micromonas pusilla*. The two putative nsLTPs found in Charophyta are all from *K. nitens*, while the remaining 27 nsLTPs identified in this work are from Chlorophyta species in which more completely sequenced genomes are accessible publicly. However, the putative nsLTPs genes are absent from the genomes of brown and red algae that we examined, including *Cladosiphon okamuranus*, *Ectocarpus siliculosus*, *Nemacystus decipiens* Onna-1, *Saccharina japonica*, and *Undaria pinnatifida* of Phaeophyta; and *Chondrus crispus*, *Cyanidiococcus yangmingshanensis*, *Cyanidioschyzon merolae*, *Galdieria sulphuraria*, *Gracilaria domingensis*, *Porphyra umbilicalis*, and *Porphyridium purpureum* of Rhodophyta. Our results showed that the putative genes could be found in the genomes of both the Chlorophyta and Charophyta species, including unicellular and multicellular algae, with the gene number of one or two per haploid genome size, except for *C.* sp. C-169, which has eight nsLTPs genes.

### Several sequence features in the algal nsLTPs are distinct from those in land plant nsLTPs

**Fig. 2:**
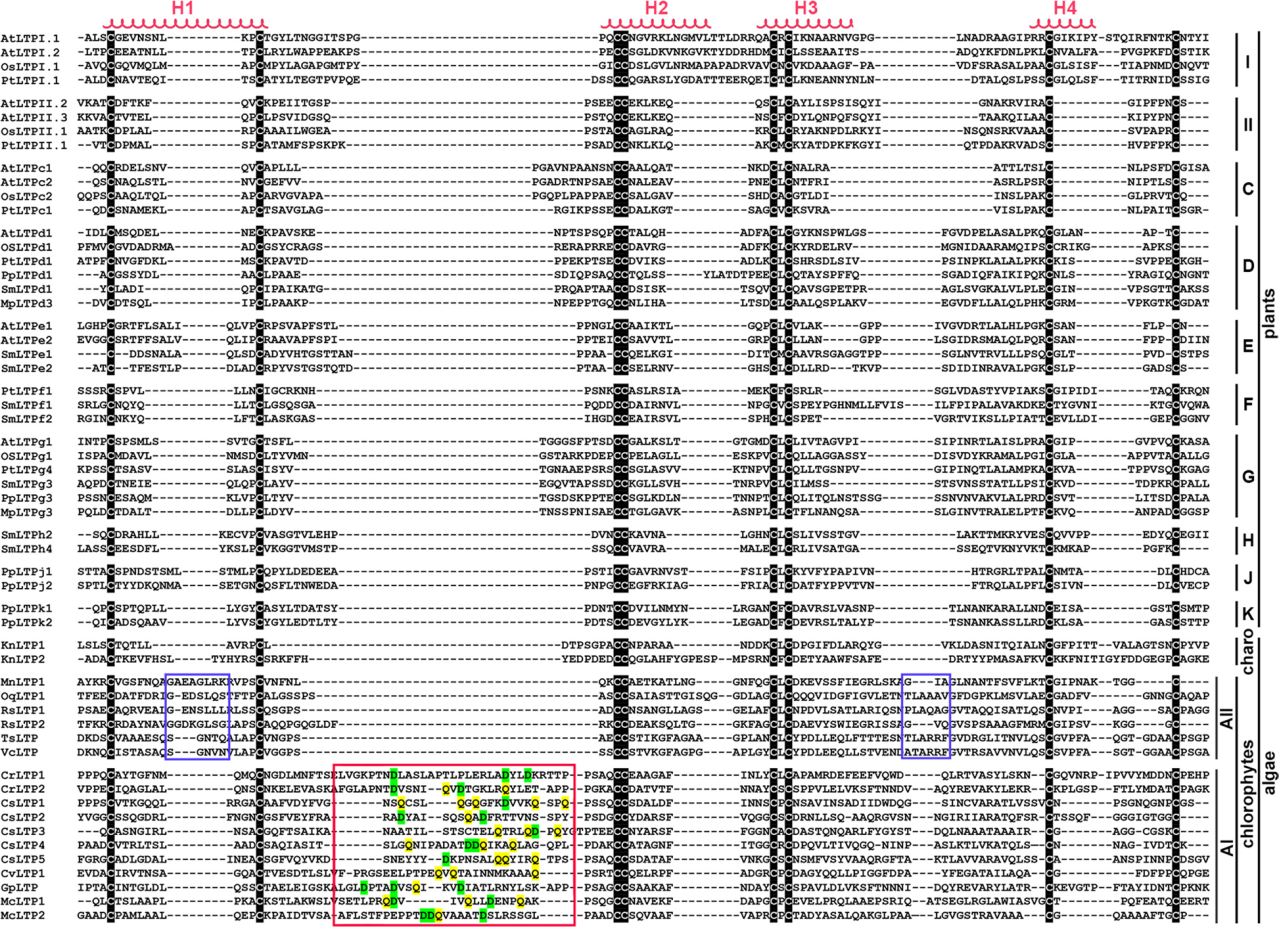
Pileup of nsLTP protein sequences from green algae and representative land plants. The sequence alignment of the 8CM region of nsLTPs from green algae and six representative land plants, including *A. thaliana*, *O. sativa*, *P. taeda*, *S. moellendorffii, P. patens*, and *M. polymorpha*, was constructed separately using MUSCLE program and piled up by nsLTP types. AI and AII denote the two algal nsLTPs subgroups of chlorophytes. In the alignment pileup, cysteine residues are highlighted and the four possible regions of a helix structure, termed H1 to H4, are marked with red spirals. The unfilled red and purple boxes respectively depict the extended loop regions that are only present in AI and AII nsLTPs. I I, II, C, D, E, F, G, H, J and K denote the nsLTP types based on the classification system proposed by Edstam et al. (2011). Charo denotes the nsLTPs of charophytes and chlorophyte denotes the nsLTPs of chlorophytes

To examine the sequence divergence of the identified nsLTPs, we used the protein sequences of nsLTPs from green algae and six representative land plants, including *A. thaliana* (dicot of angiosperm), *Oryza sativa* (monocot of angiosperm), *Pinus taeda* (gymnosperm), *Selaginella moellendorffii* (an early vascular plant), *P. patens* (a nonvascular land plant), and *Marchantia polymorpha* (the most primitive land plant). We only focused on the 8CM region for sequence comparison because most of the land plant nsLTPs either carry no extra sequences outside of the 8CM region or only contain a few residues, except for type G nsLTPs, which contain C-terminal GPI anchoring motif. For better illustration, nsLTP protein sequences of green algae and of the ten recognized types (I, II, C, D, G, E, F, H, J, and K) from the representative land plants were aligned separately with MUSCLE program and piled up. The pileup was based on the positions of the Cys residues with manual refinement according to the approximate positions of the α-helices and the loop regions. In Fig. 2, an obvious difference in the 8CM spacing sequence of the nsLTPs is presented when comparing the algal and land plant nsLTPs of which roughly two spacing patterns are in the algal nsLTPs of chlorophytes. The algal nsLTPs with an extended region in C^2^–C^3^ were further categorized into a subgroup, the AI group (“A” denotes algae), while the ones with slightly extended regions in both C^1^–C^2^ and C^6^–C^7^ were categorized into the AII group; there were relatively more members in the AI group. The nsLTPs found in *C. reinhardtii*, *C.* sp. C-169, *G. pectoral*, and *M. conductrix* were all categorized into AI nsLTPs, with an extended region enriched with hydrophilic amino acid residues, such as aspartate (D) and glutamine (Q). In silico analyses of these extended regions indicated that the AI group possesses a longer loop region linking the first helix (H1) and the second helix (H2), while the extended spacing between C^1^and C^2^ of AII nsLTPs represents a longer helical section predicted by the Helix Wheel program [41]. We did not find any unique features in the two sequences identified in *K. nitens*, which is reasonable because the information provided by merely two sequences from Charophyta is insufficient to conclude any sequence features of the nsLTP in the phylum. Moreover, the only X residue between the 5th Cys and 6th Cys (C^5^XC^6^) in the 8CM of the AII nsLTPs is leucine, a hydrophobic amino acid that is also present in other nsLTP types except for type I nsLTPs. On the other hand, proline and serine are the most frequent X residues in the C^5^XC^6^ of AI nsLTPs, which is seldom observed in the nsLTPs of land plants [32].

### The protein sequences of algal nsLTPs are located within a specific cluster in the phylogenetic tree

**Fig. 3:**
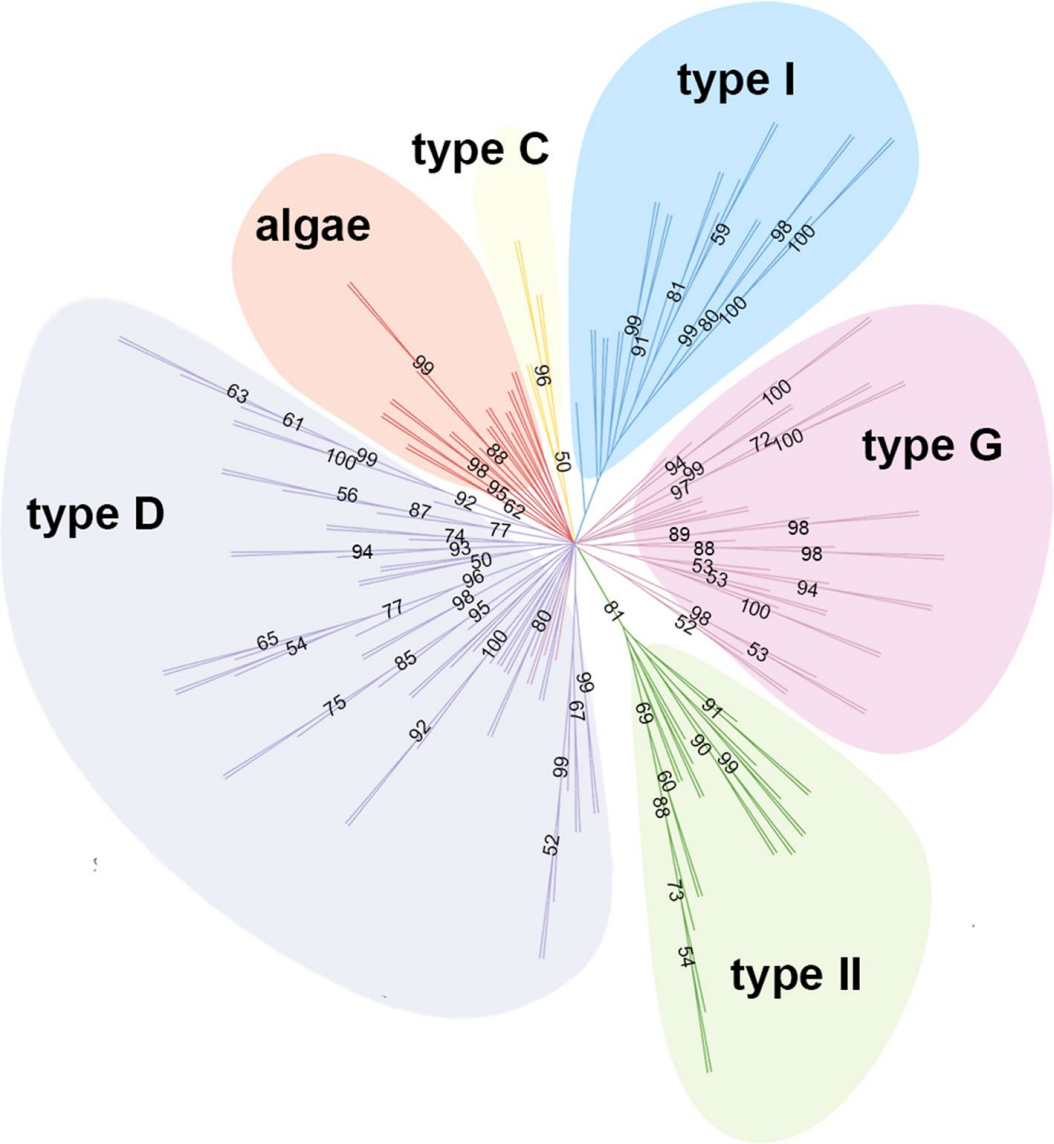
Unrooted phylogenetic tree of nsLTPs from land plants and green algae. Protein sequences of the five major nsLTP types, including type I, type II, type C, type D, and type G, from the representative land plants along with the nsLTPs found in green algae were aligned with MAFFT program in default setting. The neighbor-joining tree was constructed using the programs in PHYLIP package. Only the bootstrap values above 50 are shown on the nodes. Each color represents different nsLTP types. The algae nsLTPs in the tree are KnLTP1, KnLTP2, APLTP1, APLTP2, CrLTP1, CrLTP2, CsLTP1, CsLTP2, CsLTP3, CsLTP4, CsLTP5, CsLTP6, CsLTP7, GpLTP, McLTP1, McLTP2, MnLTP1, MnLTP2, OtLTP1, OtLTP2, RsLTP1, RsLTP2, and TsLTP. Type I nsLTPs include AtLTPI.1, AtLTPI.2, AtLTPI.3, AtLTP1.4, AtLTP1.5, AtLTP1.6, AtLTP1.7, AtLTP1.8, AtLTP1.9, AtLTP1.10, AtLTP1.11, AtLTP1.12, OsLTP1.1, OsLTP1.2, OsLTP1.3, OsLTP1.4, OsLTP1.5, OsLTP1.6, OsLTP1.9, OsLTP1.12, OsLTP1.13, OsLTP1.14, OsLTP1.16, OsLTP1.17, OsLTP1.18, and OsLTP1.20. Type II nsLTPs include AtLTP2.2, AtLTP2.3, AtLTP2.4, AtLTP2.5, AtLTP2.6, AtLTP2.7, AtLTP2.9, AtLTP2.10, AtLTP2.11, AtLTP2.12, AtLTP2.13, AtLTP2.14, OsLTP2.1, OsLTP2.2, OsLTP2.3, OsLTP2.4, OsLTP2.5, OsLTP2.6, OsLTP2.7, OsLTP2.8, OsLTP2.10, OsLTP2.11, and OsLTP2.12. Type C nsLTPs include OsLTPc1, OsLTPc2, AtLTPc1, AtLTPc2, AtLTPc3, and AmboLTPc. Type D nsLTPs include AtLTPd1, AtLTPd3, AtLTPd4, AtLTPd5, AtLTPd6, AtLTPd7, AtLTPd8, AtLTPd9, AtLTPd10, AtLTPd12, OsLTPd1, OsLTPd2, OsLTPd3, OsLTPd4, OsLTPd5, OsLTPd6, OsLTPd7, OsLTPd8, OsLTPd9, OsLTPd10, OsLTPd12, PpLTPd1, PpLTPd3, PpLTPd4, PpLTPd5, PpLTPd7, PpLTPd8, PpLTPd10, PpLTPd11, PpLTPd12, PpLTPd13, PpLTPd14, PpLTPd15, PpLTPd16, PpLTPd17, PpLTPd18, PpLTPd19, PpLTPd20, PpLTPd22, SmLTPd3, SmLTPd4, SmLTPd5, SmLTPd6, SmLTPd7, SmLTPd9, SmLTPd10, SmLTPd12, SmLTPd13, SmLTPd16, MpLTPd1, MpLTPd2, MpLTPd3, MpLTPd4, MpLTPd5, MpLTPd6, MpLTPd7, MpLTPd8, MpLTPd9, MpLTPd10, MpLTPd11, and MpLTPd12. Type G nsLTPs include AtLTPg1, AtLTPg2, AtLTPg3, AtLTPg4, AtLTPg7, AtLTPg8, AtLTPg9, AtLTPg10, AtLTPg11, AtLTPg13, AtLTPg14, AtLTPg15, AtLTPg19, AtLTPg23, AtLTPg24, AtLTPg26, AtLTPg29, AtLTPg31, OsLTPg1, OsLTPg2, OsLTPg4, OsLTPg7, OsLTPg9, OsLTPg10, OsLTPg12, OsLTPg13, OsLTPg14, OsLTPg15, OsLTPg16, OsLTPg18, OsLTPg22, OsLTPg24, PpLTPg3, PpLTPg6, PpLTPg9, PpLTPg11, PpLTPg12, SmLTPg3, SmLTPg5, SmLTPg7, SmLTPg8, MpLTPg1, MpLTPg2, MpLTPg3, and MpLTPg4

According to the previous comparison, the sequence features of nsLTPs in green algae are divergent from those of nsLTPs identified in land plants, including the 8CM spacing and the residue properties. For further confirmation, the protein sequences of nsLTPs from green algae and from the five major recognized types, I, II, C, D, and G, were tested in phylogenetic analyses with a tree constructed using the Neighbor-Join (NJ) method, which clustered neighboring sequences in a stepwise manner and was supported by 10,000 bootstrap replicates (Fig. 3). Although the nsLTPs of green algae did not form a separate clade supported with high bootstrap values, they were located to a specific cluster in the tree, indicating that the protein sequences of the algal nsLTPs are divergent from those of the established major nsLTP types in land plants, hence, could be categorized into a unique nsLTP lineage. This result is consistent with the alignment pileup in which the newly identified algal nsLTPs demonstrate different sequence features. However, we also noted that the sequences of AI and AII nsLTPs could not form two distinct mono-clades in the tree, indicating that either the members of these two algal nsLTP groups still share sequence similarity or simply because the number of AII nsLTPs for the analysis is not sufficient to form a new clade. In addition, some of the type G nsLTPs were clustered into type D, including SmLTPg8, MpLTPg1 and MpLTPg4, which might be due to a close evolutionary relationship for the Type D and G nsLTPs (Fig. S1).

### The range of distributions of the molecular mass and isoelectric point of the nsLTPs in green algal are different from those of the land plant nsLTPs

**Fig. 4:**
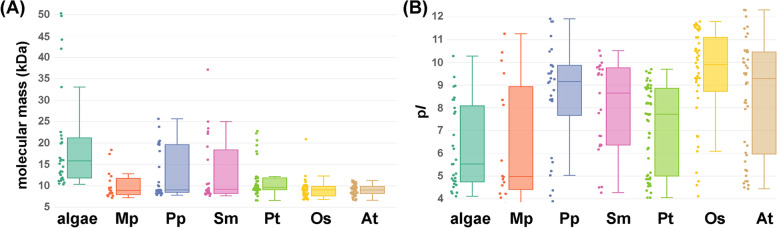
Distribution patterns of the molecular mass and p*I* value of the nsLTPs from green algae and the representative land plants. The molecular mass and theoretical p*I* value of each nsLTP in the following organisms were demonstrated in the boxplots **(A)** and **(B)**, including the investigated species of green algae (algae), *M. polymorpha* (Mp), *P. patens* (Pp), *S. moellendorffii* (Sm), *P. taeda* (Pt), *O. sativa* (Os), and *A. thaliana* (At). The color dots beside the boxplot represent the nsLTP molecular mass or the p*I* value, and the horizontal lines of the boxplot from bottom to top mark the lower inner fence, the first quartile, the medium, the third quartile, and the upper fence

Plant nsLTPs are a group of small proteins, mostly with a molecular mass ranging from 6 to 10 kDa and isoelectric point (p*I*) ranging from 8 to 12. To determine if the algal nsLTPs also possess similar properties, we analyzed the theoretical molecular masses and p*I* values of the algal nsLTPs. In comparison, we also computed and analyzed the nsLTPs of six land plant species, including two nonvascular bryophytes: *M. polymorpha* and *P. patens* and four vascular plants: S*. moellendorffii*, *P. taeda*, *A. thaliana*, and *O. sativa*. Type G nsLTPs were not included in the analysis owning to the possession of a long GPI anchoring region at the C-terminal end. Thus, respectively, 25, 16, 29, 23, 54, 46, and 44 nsLTPs from green algae, *M. polymorpha* (a liverwort), *P. patens* (a moss), *S. moellendorffii*, *P. taeda*, *O. sativa,* and *A. thaliana* were used in the following analyses. To compare the molecular mass and p*I* of the nsLTP from green algae and those from land plants, the analyses results were shown as boxplots. In Fig. 4A, the molecular mass of algal nsLTPs lays in the range of 10.36–50.28 kDa; in contrast, the molecular mass of nsLTP falls in the range of 7.23–18.4 kDa, 7.82–25.66 kDa, 7.67–37.15 kDa, 6.66–22.80 kDa, 6.82–20.89 kDa, and 6.64–11.27 kDa in *M. polymorpha*, *P. patens*, *S. moellendorffii*, *P. taeda*, *O. sativa*, and *A. thaliana*, respectively. In summary, molecular masses of all nsLTPs in green algae are greater than 10 kDa, mostly in the range of 10–25 kDa (82.76%). However, only 30–40% of the nsLTPs in bryophytes, including *M. polymorpha* and *P. patens*, have a molecular mass greater than 10 kDa. The nsLTP molecular mass distributed to a smaller value is more obvious in the two angiosperms. That is, only 19.57% of *O. sativa* nsLTPs and 18.18% of *A. thaliana* nsLTPs have a molecular mass greater than 10 kDa. To conclude, unlike the land plant nsLTPs, which a majority have a molecular mass less than 10 kDa, the nsLTPs in green algae are of a greater molecular mass.

Likewise, the nsLTPs in green algae, *M. polymorpha*, *P. patens*, *S. moellendorffii*, *P. taeda*, *O. sativa*, and *A. thaliana* have p*I* falling in the range of 4.11–10.28, 3.8–11.26, 3.89–11.92, 4.27–10.52, 4.05–9.70, 4.11–11.80, and 4.44–12.31 (Fig. 4B). The p*I* values of the nsLTPs in each species fall in a broad range with an increasing trend from the species of green algae to vascular plants. The nsLTPs in moss and the vascular land plants generally have a basic p*I* with the value above 8. More specifically, 75.86, 52.17, 44.44, 80.43, and 65.91% of the nsLTPs in *P. patens*, *S. moellendorffii*, *P. taeda*, rice, and Arabidopsis, respectively, exhibit a p*I* value above 8. On the other hand, instead of clustering above 8, the p*I* values of the nsLTPs in green algae and liverwort distribute more discretely and majorly exhibit low p*I* value; that is, there are only 24.14% of the algal nsLTPs and 37.50% of the nsLTPs in *M. polymorpha* with a p*I* value above 8.

### The two putative nsLTPs genes identified in Chlamydomonas both carry multiple introns and are adjacent to each other in head to tail fashion

**Fig. 5:**
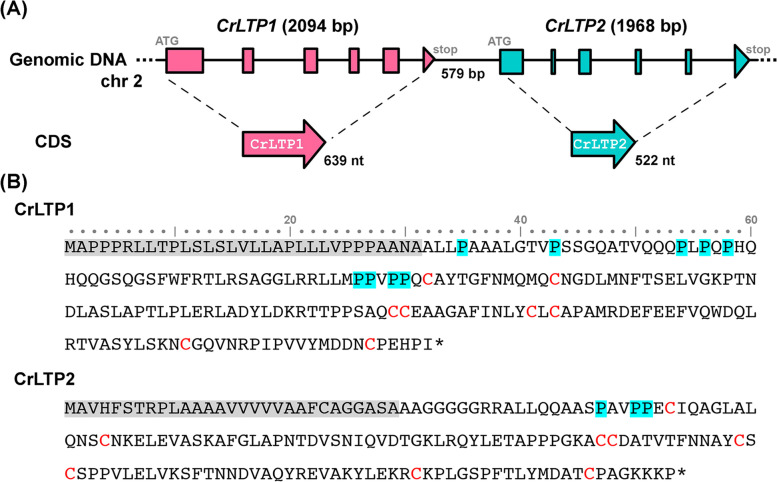
Gene and protein features of CrLTPs. **A** Gene features of *CrLTP1* and *CrLTP2*. The schematic diagram depicted the orientation and exon/intron distributions of *CrLTP1* (pink) and *CrLTP2* (green) genes. The length of the two genes, the region in between, and the coding region sequence (CDS) are illustrated. Exons are depicted as color-filled boxes and arrowheads, and introns as solid lines. **B** Amino acid sequences of CrLTP1 and CrLTP2. The secretory signal peptides (SPs) are shaded with grey, the proline residues in the N-terminal region upstream of the first cysteine residue are highlighted with blue shade, and the cysteine residues of the 8CM are marked in red

From the results of previous analyses, including the phylogenetic analysis and the distribution patterns of the molecule mass and p*I* values, the algal nsLTPs identified in this study could be considered a new lineage of nsLTPs. To further investigate the algal nsLTP properties, such as the characteristics of the genes, transcripts, and protein, as well as the in vitro functions, we performed analyses on the two nsLTPs identified in *C. reinhardtii*, termed CrLTP1 and CrLTP2. The genes of *CrLTP1* and *CrLTP2*, which orient to the same direction, are respectively 2094 and 1968 base-pairs in length and are located 579 base-pairs apart on chromosome II (Fig. 5A). Unlike the land plant nsLTP genes, which mostly have only one intron after the 8th Cys codon (C^8^), the *CrLTPs* and a majority of other algal nsLTP genes carry multiple introns (Table S1). The relative positions of the *CrLTP1* and *CrLTP2* introns are also depicted in Fig. 5A. Furthermore, the *CrLTP1* and *CrLTP2* transcripts are 639 and 522 nucleotides, respectively; the former encodes a protein of 212 amino acid residues, 31 residues of which are predicted as SPs. The molecular mass of the mature CrLTP1 was 19.9 kDa, and the theoretical p*I* value was 5.53. The transcript of *CrLTP2*, on the other hand, encodes protein of 173 amino acid residues, 29 residues of which are predicted as SPs (Fig. 5B). The molecular mass and p*I* value of the CrLTP2 protein were 15.2 kDa and 8.64, respectively. In addition, an extra N-terminal region of 60 and 23 amino acid residues in CrLTP1 and CrLTP2 lie upstream of the first Cys residue of the 8CM; this region is often not present in the plant nsLTP. The N-terminal region of CrLTP1 is enriched with glutamine, Q (16.7%), proline, P (15.0%), and leucine, L (13.3%) and that of CrLTP2 is enriched with alanine, A (26.0%), glycine, G (21.7%), and proline, P (13.0%) (Fig. 5B). Pro and Gly are considered turn-forming residues; together, they contribute 25.0 and 34.8% in the N-terminal region of CrLTP1 and CrLTP2, respectively.

### *CrLTPs* expressed majorly and specifically in the late zygote stage

**Fig. 6:**
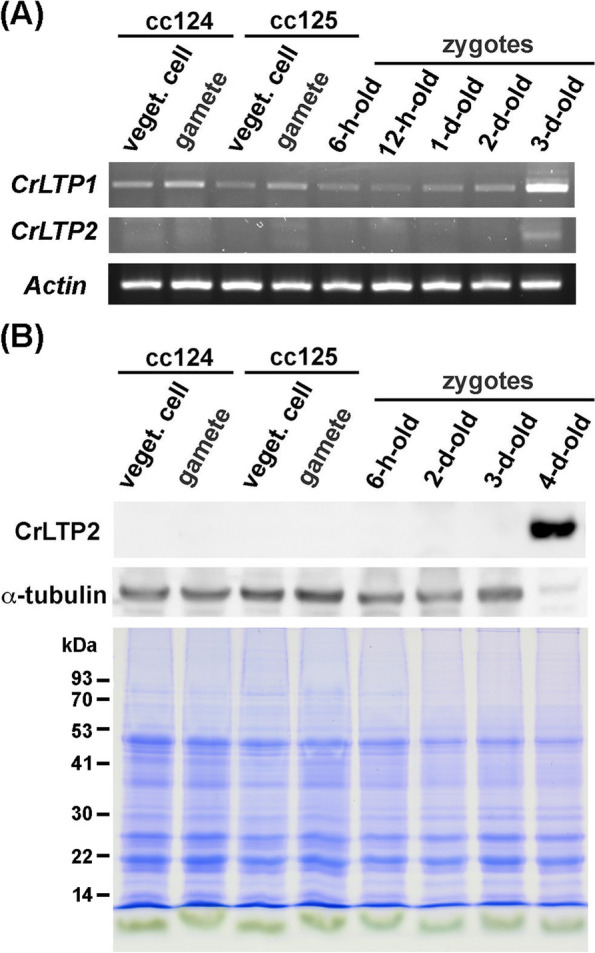
The expression patterns of CrLTPs transcripts and proteins in different developmental stages of Chlamydomonas. **A** RT-PCR analysis of the transcript levels of *CrLTP1* and *CrLTP2* in different developmental stages of Chlamydomonas, including the vegetative cells and gametes of both mating types, cc124 (mt^−^) and cc125 (mt^+^), and zygotes collected 6 hours, 12 hours, 1 day, 2 days and 3 days after mating. Actin was used as a normalization control. The displayed regions were cropped from three agarose gels loaded with the RT-PCR products using primers sets specific to *CrLTP1, CrLTP2* and *Actin*. The full length gels are presented in Supplementary Fig. 5. **B** The expression pattern of CrLTP2 protein in Chlamydomonas. Total proteins of vegetative cells and gamete of both strains, and zygote cells collected from different time points *after gamete mating* were resolved with SDS-PAGE and visualized by immunoblotting against CrLTP2. Immunoblotting against α-tubulin was used as a quantity reference. A replicate gel stained with Coomassie brilliant blue was also provided as another reference of the loading control due to an unknown factor that abruptly weakened the signal of α-tubulin in the 4-d-old zygotes. The molecular masses are indicated at left in kDa. The displayed regions were cropped from the images captured from the same PVDF membrane blotted with CrLTP2 and α-tubulin antibodies. The full length blots and gel are presented in Supplementary Fig. 6

To investigate the expression patterns of *CrLTP1* and *CrLTP2*, we analyzed the gene transcript levels in different developmental stages using the transcriptome data of *C. reinhardtii* provided by Dr. Huang in UC Riverside, USA [42]. As shown in Supplementary Fig. 2, both *CrLTP1* and *CrLTP2* express specifically in the zygote and tetrad stages. The RPKM (Reads Per Kilobase per Million mapped reads) value of *CrLTP1* shifts above 200 in the zygote stage and is above 60 in the tetrad stage, while the RPKM value of *CrLTP2* is only 6 and 10 in the zygote and tetrad stage, respectively; both transcripts are barely detected in the vegetative and gamete cells (Fig. S2). Based on these results, we collected the total RNA and protein lysates of the Chlamydomonas cells from the stages before and during zygotes development and further analyzed the expression patterns of *CrLTP*s using RT-PCR and immunoblotting. As shown in Fig. 6A and Supplementary Fig. 5, the expression of both *CrLTP1* and *CrLTP2* peak in the stage of 3-d-old zygotes compared to other cell types and zygote stages. However, upon closer inspection, signals of a small-scale expression of *CrLTP1* were detectable in all cell types and zygote stages, while *CrLTP2* was only detected in 3-d-old zygotes.

As for protein expression analysis, the creation of antibodies against both CrLTPs was attempted. However, due to difficulties in protein purification, the antibody against CrLTP1 generated by a short-peptide-based approach did not react well and was discarded. Therefore, for the following examination of protein detection, we only used the antibody against CrLTP2 generated by whole protein induction, which presents acceptable sensitivity and specificity (Fig. S4). As shown in Fig. 6B and Supplementary Fig. 6, the timing that is of the highest CrLTP2 protein level was slightly delayed by 1 day compared to that of the highest transcript level, which peaks at the 4-d-old zygote stage. No protein signals were detected in the vegetative cells, gametes, and the young zygotes.

### The purified CrLTP2 protein demonstrated a solid affinity to long chain fatty acids and displayed resistance to protease activity in vitro

**Fig. 7:**
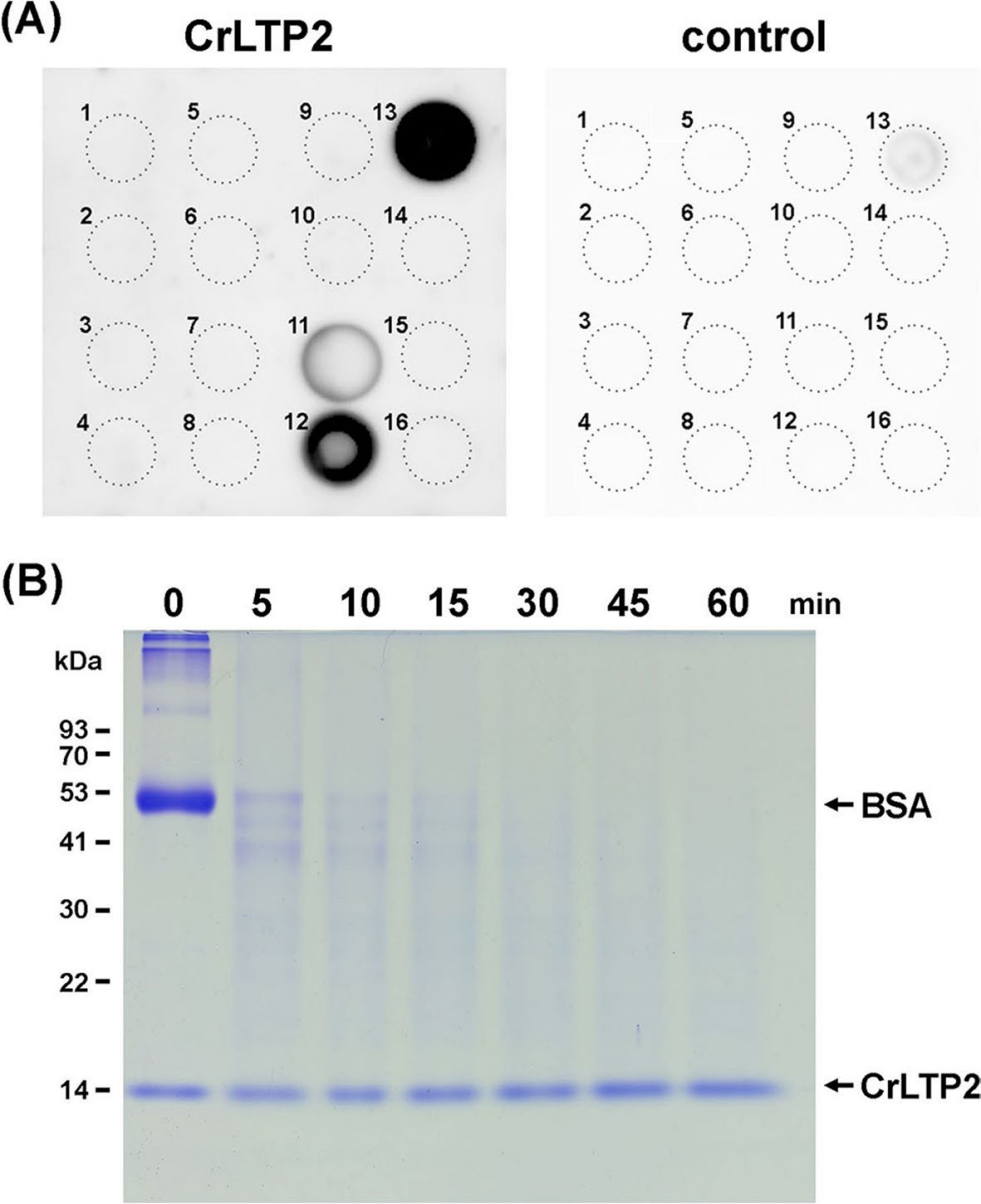
Protein-lipid overlay (PLO) assay and proteinase digestion analysis of CrLTP2. **A** PLO assay. The binding ability of CrLTP2 protein to the pre-spotted lipid materials were tested. A membrane dotted with the lipid ligands and a duplicated control membrane are respectively reacted with the purified CrLTP2 protein (CrLTP2) and without the protein (control) and immunodetected with an anti-CrLTP2 antibody. The dotted circles depict the position of the pre-spotted lipid materials, as indicated: 1, Phenylalanine; 2, cinnamic acid; 3, p-coumaric acid; 4, caffeic acid; 5, ferulic acid; 6, capric acid (C10:0); 7, lauric acid (C12:0); 8, myristic acid (C14:0); 9, palmitic acid (C16:0); 10, stearic acid (C18:0); 11, arachidic acid (C20:0); 12, behenic acid (C22:0); 13, lignoceric acid (C24:0); 14, 12-hydroxystearic acid; 15, 15-hydroxypentadecanoic acid;16, 16-hydroxyhexadecanoic acid. **B** The analysis of the protease-resistant ability of CrLTP2 protein. Bovine serum albumin (BSA), the control substrates, and CrLTP2 protein were mixed, incubated with protease and collected at different time points: 0, 5, 10, 15, 30, 45, and 60 minutes. The digested protein products were resolved using SDS-PAGE and stained with Coomassie Brilliant Blue dye. Cropped blot images are shown, and full length blots are presented in Supplementary Fig. 7. The numbers on the left indicate the protein molecular masses in kDa, and the arrows on the right indicate the positions of BSA and CrLTP2

To analyze protein properties, we started by cloning the *CrLTP1* and *CrLTP2* genes and fused them to thioredoxin (*Trx*) to produce recombinant proteins with correct folding and better solubility. However, using the Origami B strain of the *E. coli* system to overexpress these cysteine-rich proteins still led to insoluble protein accumulation and thus resulted in very low productivity of both *Trx-CrLTP1* and *Trx-CrLTP2* encoded proteins. From the sequence analysis, the N-terminal of both CrLTPs are enriched with Pro and Gly, the turn-forming residues, which might decrease the protein solubility owning to a slower folding process [43]; that is, the N-terminal region of both CrLTPs might have some unknown effects on the efficiency of CrLTP protein purification. Hence, we removed the N-terminal region of CrLTPs that is enriched with Gly and Pro to circumvent the purification problem in the *E. coli* system. Because only the CrLTP2 protein was successfully purified on this modified procedure, the generation of the CrLTP2 antibody, and the following analyses were thus conducted using the purified CrLTP2 protein (Fig. S3). To examine the lipid-binding ability of CrLTP2, the protein-lipid overlay (PLO) assay was conducted with different lipid samples, including hydroxyl and saturated fatty acids with different carbon chain lengths (C10:0–C24:0), phenylalanine, and several cell wall precursors [44]. In Fig. 7A, the signals of CrLTP2 protein were only detected on the spots of very long-chain fatty acids, including arachidic acid (C20:0), behenic acid (C22:0), and lignoceric acid (C24:0). No signals were detected in other compounds, including those serving as precursors of cell barriers: hydroxyl and short-chain fatty acids, phenylalanine, cinnamic acid, p-coumaric acid, caffeic acid, and ferulic acid. In addition, the signal was enhanced with the increasing of the fatty acid alkyl chain length. Hence, the PLO assay demonstrated that the algal nsLTP, CrLTP2, can bind to lipid molecules, such as the saturated fatty acids, of which the affinity is increased by the chain length of the fatty acids from C20:0 to C24:0 (Fig. 7A).

We also conducted a protease digestion assay to test if CrLTP2 possesses resistance to protease ability, which is a common feature of land plant nsLTPs. Equal amounts of the purified CrLTP2 protein and the control substrate, bovine serum albumin (BSA), were mixed and incubated with a serine protease (Pronase E) and were collected at different time points for SDS-PAGE analysis. As shown in Fig. 7B, unlike the signals of BSA that instantly dropped in five minutes of the protease incubation until being undetectable in 45 minutes, the signals of CrLTP2 protein remained the same, indicating that the CrLTP2 protein is protease-resistant.

## Discussion

Previously, it has been shown that nsLTPs were widely distributed in the plant kingdom from basal bryophytes to advanced angiosperms and played several roles in plant adaptations during their migration to the land environment [45]. However, their existence prior to that of the most primitive land plants has not been identified [32]. In this study, we found nsLTPs in both chlorophytes and charophytes of green algae but not in the investigated species of red and brown algae, which indicates that the earliest nsLTPs may have risen from a common ancestor of green algae and land plants. In addition, the gene number of nsLTPs in the investigated green algae is mainly one or two per haploid genome, which is comparably fewer than those of the land plants. This observation leads to speculation that the earliest nsLTP occurred in the common ancestor of green algae and land plants before turning into a multigene family with dozens of members in land plants; that is, the gene numbers of nsLTPs kept growing in non-vascular and vascular plants after plants had adapted to the terrestrial environment. However, there were exceptions in our search because no nsLTP were identified in ten of the 23 investigated green algae species listed in Table 1, thus requiring further investigation. At this stage, we can only speculate that it might be due to the fragmental and incomplete genome sequencing, weak sequence similarities, or simply because the function of nsLTPs is not essential for the survival of these green algae species. In addition, the enlargement of the gene family during plant evolution may not only be positively correlated to plant cell differentiation into different cell types, tissues, and functional organs but also to the enhanced ability of plants to cope with the biotic and abiotic stresses from the harsh land environment [18, 38, 46].

The nsLTP genes in green algae carry multiple introns located among the coding sequences of the 8CM region; for example, the one nsLTP gene found in *G. pectoral* carries four introns, both *CrLTP1* and *CrLTP2* carry five introns, and the nsLTP gene in *Tetrabaena socialis* carries six introns (Table S1). On the contrary, most of the land plant nsLTP genes either have only one intron located downstream of the last Cys (C^8^) codon in the 8CM or have none. Since the intron number and position of land plant nsLTPs is one of the important criteria in the classification system of nsLTP types [31, 32]; it makes the algal nsLTPs a very distinctive lineage of nsLTPs for carrying multiple introns located in the 8CM region. Since the nsLTP genes in land plants, which increased substantially in gene numbers, have much fewer introns, we also speculated that the duplication of nsLTP genes should occur after their intron number reduction. Moreover, a recent study suggested that intron-less genes occurred later than intron-rich genes and were subjected to higher selection pressure and stronger functional constraints [47]. Accordingly, the existence of land plant nsLTPs with fewer intron numbers, in contrast to the intron-rich nsLTPs in green algae, may be the result of purifying pressures from the processes of plants adapting to the harsh land environment. Because the intron-rich genes implied a more complicated gene duplication process if compared to the intron-poor genes, the massively increased gene numbers of the nsLTPs in land plant species could also be caused by losing the multiple-introns feature carried by algal nsLTPs [48].

Type I pairing and type II pairing are the two pairing patterns of nsLTP disulfide bonds; the former is linked by C^1^-C^**6**^, the C^**5**^-C^8^, C^2^-C^3^, and C^4^-C^7^, and the latter is linked by C^1^-C^**5**^, the C^**6**^-C^8^, C^2^-C^3^, and C^4^-C^7^, instead. These two pairing patterns could be judged by the amino acid properties of the X residue of C^**5**^XC^**6**^ [49, 50]. The X in type I pairing is a hydrophilic amino acid facing the surface of the protein core; this pairing pattern appears in most type I nsLTPs but few in other types. Whereas the type II pairing pattern in which a bulky and non-polar X residue at the center of C^5^XC^6^ projects toward the hydrophobic cavity is displayed by the remaining nsLTP types, including type G and D of the earliest land plants [51, 52]. In the evolutionary history of the nsLTP family, the type II pairing pattern may appear earlier that the type I pairing pattern, which is present in the vesicular land plants starting from ferns [32]. In this research, we found that the majority of X located between C^5^ and C^6^ of the algal AI nsLTPs are proline (P) and serine (S), both of which are natural amino acids with small volumes. Serine possesses a slightly polar property that could provide hydrogen bonds. These two residues are rarely observed in the C^5^XC^6^ of land plant nsLTPs, apart from two type I nsLTPs found in *O. sativa*. However, due to the 3D structures of neither type I nsLTPs being available, it is difficult to draw comparisons to the pairing patterns of AI nsLTPs. However, in the AII nsLTPs and the *Klebsormidium* nsLTPs, leucine (L) is the only X residue of C^5^XC^6^, which indicates the possible existence of type II pairing patterns in green algae nsLTPs (Fig. 2).

Unlike most plant nsLTPs, which underwent a decline in molecular mass during evolution, algal nsLTPs had retained its greater molecular mass. Type AI nsLTPs possess a longer sequence between C^2^ and C^3^ which is predicted by the Helix Wheel program as a loop structure linking the first two possible α-helices, H1 and H2, with a significant portion of charged amino acid residues D and Q (Fig. 2). This loop section may render flexibility to nsLTP central cavity folded by helices and linked by loops or lead some roles in the interaction of the algal nsLTPs with other proteins or compounds on the way to their extracellular destinations. In addition, the extra N- and C- terminal regions that flank the sequence of 8CM of the algal nsLTPs are another factors that increase the molecular mass. Unlike the 8CM region, which is responsible for core structure formation and ligand binding, these terminal regions may serve some roles in protein-protein interactions or post-translational protein modification, which will be mentioned in the next section.

From previous analyses, both Chlamydomonas nsLTPs, CrLTP1 and CrLTP2, shared high degrees of protein sequence similarity and overlapping expression patterns, implying possible functional redundancy. Nevertheless, due to the adjacent genomic positions of *CrLTP1* and *CrLTP2*, it is very difficult to create double knockout mutant lines for protein functional analysis in vivo; therefore, the following speculation was made regarding the roles played by algal nsLTPs based on the in vitro analysis of CrLTP. It has been shown that the purified CrLTP2 could bind fatty acids with C20, C22, and C24 aliphatic chains in PLO assay; this, combined with the elevated CrLTP2 protein levels at the late zygotic stage and the thickened cell walls, indicates that there may be lipid-related materials being bound and transported by CrLTP2 within the zygote cell walls [53]. Indeed, there are several physiological functions of nsLTPs proposed thus far, yet a significant portion of which are linked to the hydrophobic materials associated with the cell wall, such as cutin, suberin, and sporopollenin [23–25]. Nevertheless, instead of cellulose, the zygotic cell walls of *C. reinhardtii* are mainly composed of the extensin-like hydroxyproline-rich glycoproteins (HRGPs), carrying numerous proline residues for hydroxyl modification, which could conjugate with saccharides for glycoproteins synthesis and cross-link to each other to provide the adhesion property of the thicken zygotic walls [54–56]. In *C. reinhardtii*, both CrLTPs possess the proline-rich N-terminal region. Although the amino acid residues in this region are not arranged as repeated XP or XP_3–5_ motifs that were later modified into hydroxyproline as a typical member of the cell wall HRGPs family, these proline residues were predicted to be susceptible for further hydroxylation if analyzed in silico (data not shown) [57, 58]. However, further investigation is required before assuming that CrLTPs could cross-link to each other or to other components and render functions to Chlamydomonas zygotic cell walls. In addition, although the mechanism of how lipid materials participate in the algal wall synthesis has not been elucidated, a sporopollenin-like algaenan layer composed of lipid-derived nonhydrolyzable materials was identified in the Chlamydomonas genus as one of the multiple layers in the enhanced zygotic cell wall during zygospore maturation [59]. A similar material that was identified in *Chlamydomonas monoica* is composed of aliphatic polymers of hydroxylated long-chain fatty alcohols and fatty acids ranging from C22:0 to C30:0 with approximately 24 carbon chain on average [59, 60]. Even though this layer has not been reported in *C. reinhardtii*, an essential role of long-chain aliphatic polyketide building blocks for a durable zygospore wall has been proposed based on the identification of a type I polyketide synthase, PKS1 [61]. This enzyme was implicated in the synthesis of the knob-like structure at the cell surface and the central wall layer of the Chlamydomonas zygotes, mutation of which severely affected the resistance ability of the zygospores to the environmental stresses. In the report, the levels of PKS1 transcripts reached a maximum around 48 hours after gametes matting, which is exactly 1 day earlier than the surge of both CrLTPs transcript levels and 2 days earlier than the signal of CrLTP2 protein is detected in the zygotes (Fig. 6). The temporal expression patterns of CrLTPs and PKS1 also intensify the previous speculation of the existence of the aliphatic material that is bound to CrLTP proteins during zygote development in *C. reinhardtii*. We need to investigate more on this topic to confirm this hypothesis. In conclusion, the observations of the CrLTPs expression patterns, the long-chain fatty acids binding, and the protease-resistance abilities suggested that the functions of algal nsLTP should be very similar to their counterparts in land plants.

## Conclusion

In this report, we identified putative nsLTP genes in several species of green algae and investigated their properties. We found that the algal nsLTPs, unlike those in land plants, carry an extended spacing sequence in the region of 8CM, which is enriched with hydrophilic residues, and possess extended sequences at both terminals flanking the 8CM with turn-forming residues. Moreover, the results of further examination of the CrLTPs in Chlamydomonas, including the presence in the late zygotic stage, the temporal expression patterns and the lipid-binding ability, shed light on the function of algal nsLTPs and implied the possible existence of lipid-derived components in the Chlamydomonas zygotic cell walls. These characteristics make the algal nsLTPs a distinct lineage in the nsLTPs family. This work expanded the landscape of our understanding about the nsLTPs family, including their distributions in the evolutionary lineage of plants and the potential functions in the zygotic cell walls. In the future, the physiological roles and the possibility of nsLTPs constituting and interacting with the components of algal cell walls should also be explored.

## Methods

### In silico identification of nsLTPs in algae

The data mining used databases of the algal genomes with complete sequences from the National Center for Biotechnology Information (NCBI, https://www.ncbi.nlm.nih.gov/) and from the Joint Genome Institute (JGI, https://phytozome-next.jgi.doe.gov/). Putative nsLTPs were identified by surveying the genomes of different algae species using the conserved 8CM sequence (C-X_n_-C-X_n_-CC-X_n_-CXC-X_n_-C-X_n_-C) as the query with a Perl script. The X_n_ indicate any amino acids ranging from 8 to 50 residues. The potential candidates that possess the 8CM sequence arrangement with acceptable spacing residue number were further analyzed with a signal peptide prediction program, SignalP 5.0 (http://www.cbs.dtu.dk/services/SignalP/). These SPs and 8CM-containing proteins were further subjected to motif analysis using a bioinformatics tool provided by GenomeNet (https://www.genome.jp/tools/motif/) based on the Hidden Markov Model (HMM). Only those with the LTP2 motif or without any annotated motifs from the Pfam database were considered nsLTPs.

### Prediction of sequence features, isoelectric points and molecular masses

The number of introns and their positions of the putative nsLTP genes was obtained from the fully sequenced algal genomes of interest. The potential GPI anchor post-transcriptional modification site was predicted using an online software predGPI (http://gpcr2.biocomp.unibo.it/predgpi/pred.htm). As for the protein molecular mass and isoelectric point (p*I*) analysis, the sequences of nsLTPs without SPs from the algal species and five land plants, including *Physcomitrella patens* (a non-vascular plants), *Selaginella moellendorffii* (a basal vascular plant), *Pinus taeda* (a gymnosperm), *Oryza sativa* (a monocot of angiosperms), and *Arabidopsis thaliana* (a dicot of angiosperms) were computed using the package Bio.SeqUtils of Biopython. Cys residues that form the four disulfide bonds in the 8CM were omitted from the calculation.

### Multiple sequence alignment and phylogenetic tree construction

For the relationships of the 8CM containing proteins identified in plants, the mature protein sequences of 11 putative algal nsLTP candidates and the representative members in 8CM-containing families of land plants, including cereal amylase trypsin inhibitors (ATI), 2S albumins, hybrid proline-rich proteins (HyPRP) and nsLTPs were subjected to multiple sequence alignment driven by MAFFT program with default settings [39, 40]. The tree was constructed using a series of programs starting from Seqboot, Protdist, Neighbor, to Consense in PHYLIP package version 3.698 according the distance matrix of PAM model and was supported by a bootstrap test of 1000 re-samplings [62]. The alignment pileup in Fig. 2 is constituted by the protein sequence alignments from the ten nsLTPs types in the representative land plants, including *A. thaliana*, *O. sativa*, *P. taeda*, *S. moellendorffii, P. patens*, and *M. polymorpha*, and the newly identified algal with manually refinement; the nsLTPs of each type was aligned separately using the MUSCLE program [63]. Protein sequences of type I, type II, type C, type D, and type G nsLTPs from the representative land plants, including *P. patens*, *S. moellendorffii*, *O. sativa*, and *A. thaliana*, and the algal nsLTPs sequences were used in the phylogenetic analysis, of which the signal peptide and the sequence after the GPI-anchor modification site were removed. After the multiple sequence alignment were produced using MAFFT program with default settings, a neighbor-joining tree was constructed using the programs in the PHYLIP package with default setting. Distance matrices were computed from the aligned nsLTP sequences using PRODIST program based on the PAM substitution matrix, followed by using NEIGHBOR program with default settings to build a phylogenetic tree. The final tree was supported by a bootstrap test of 10,000 re-samplings from the origin dataset and viewed in Seaview program [64].

### *Chlamydomonas reinhardtii* culture and zygotes generation

The vegetative cells, including CC-124 (mt^−^) and CC-125 (mt^+^), were separately maintained in Tris-Acetate-Phosphate [65] agar medium in an incubator at 25 °C under a 16-h-light/8-h-dark cycle and light intensity of 3000 lx [66]. For gametes induction, the vegetative cells that were grown in TAP medium with continuous sharking at 80 r.p.m. for 3 days to reach 1*10^6^ cells/ml cell density were transferred into nitrogen depleted TAP medium and cultured for another 3 hours to produce gamete cells. Equal amounts of the cells from both gamete strains were mixed and kept under light at 25 °C for 1–2 hours without shaking for the gametes to mate and generate zygotes that are maintained in the nitrogen-depleted TAP medium in dark for future use.

### RNA extraction and RT-PCR analysis

Vegetative cells, gametes, and the zygotes that were collected different time points at 6 hours, 2 days, 3 days, and 4 days after the gamete mating were harvested with brief centrifugation, followed by being grounded to a fine powder in liquid nitrogen with mortars and pestles. Total RNA was extracted using Trizol reagent (Total RNA Isolation Reagent) as described by the manufacturer (Thermo Fisher Scientific, USA) and purified with an RNA isolation kit, illustra RNAspin mini (GE Health Care, USA). First-strand cDNA, which was synthesized from the total RNA using SuperScript™ III Reverse Transcriptase (Invitrogen/Thermo Fisher Scientific, USA), was used as the template for RT-PCR. The primer sets listed in Supplementary Table 2 were designed according to the target sequences and the amplified DNA fragments were separated by 1% agarose gels and visualized with an ultra-violet trans-illuminator.

### Molecular cloning of *CrLTPs* from *C. reinhardtii*

DNA fragments of *CrLTP1* and *CrLTP2* were cloned using nested polymerase chain reaction (nested-PCR) with the pool of first-strand cDNA that was generated from the purified total RNA of *C. reinhardtii* zygotic cells. As mentioned before, zygotic total RNA was extracted using Trizol reagent (Thermo Fisher Scientific, USA) and purified with an RNA isolation kit (GE Health Care, USA). The first-strand cDNA was synthesized by the SuperScript™ III Reverse transcriptase (Invitrogen/Thermo Fisher Scientific, USA); it was used as the template to generate DNA fragments of *CrLTP1* and *CrLTP2* coding sequences (CDS) by nested-PCR with two sets of PCR primer pairs (Table S2). The PCR products from first amplification with the primer sets, CrLTP1-F/CrLTP1-R and CrLTP2-F/CrLTP2-R, respectively, were used as the templates for the subsequent PCR amplification with the second primer sets, CrLTP1-nested-F/CrLTP1-nested-R and CrLTP2-nested-F/CrLTP2-nested-R. The migration pattern and sizes of the amplified DNA fragments from aforementioned nested-PCR procedure were analyzed on 1% agarose gels, and these *CrLTPs* gene fragments were sub-cloned into pGEM-T Easy vectors (Promega, USA) for DNA sequencing confirmation and served as the gene templates for the following experiments.

### Expression and purification of CrLTPs in the *E. coli* system

The fragments of *CrLTPs* coding sequence without the N-terminal part upstream of the first Cys codon were synthesized by PCR using a forward primer with the coding sequence of Tobacco etch virus (TEV) protease recognition site. The primer sets for PCR amplification in this research are listed in Supplementary Table 2. The PCR products of *TEV-CrLTP2* were cloned into a protein expression vector pET32a using the restriction sites of KpnI/XhoI for Thioredoxin (Trx) conjugation to improve the solubility of the recombinant proteins [67]. Subsequently, the pET32a*-Trx-TEV-CrLTP2* construct was transformed into an *E. coli* strain, Origami B, and selected with 100 mg L^− 1^ Ampicillin [68]. The cells were cultured at 37 °C in the LB medium until they reached an optical density at 600 nm (OD_600_) of 0.5. Then, the expression of Trx-CrLTP2 protein was induced with 1 mM isopropyl-β-D-1-thiogalactopyranoside (IPTG) at 20 °C for 16 hours. After the pellet was harvested and resuspended in buffer A (50 mM Tris, pH 7.5, 500 mM NaCl), the cells were disrupted in the presence of protease inhibitors using sonication, and the supernatant was loaded onto a nickel-nitrilotriacetic acid (Ni-NTA) agarose column and then washed with buffer A containing 20 mM imidazole. The Trx-CrLTP2 fusion protein was eluted by buffer A containing 250 mM imidazole. After the elution, 6xHis-tagged TEV protease was used to release CrLTP2 form bound Thioredoxin (4 °C, overnight). The residual uncleaved Trx-CrLTP2 protein and 6xHis-tagged TEV protease can be further removed through subtractive Ni-NTA purification. Protein samples were resolved by denaturing SDS-PAGE to examine the purity and determine whether the purified protein was of the expected size.

### Antibody preparation, SDS-PAGE, and immunoblotting analysis

To prepare a suitable antibody for CrLTP2 probing, the purified CrLTP2 protein was sent for antibodies synthesis. The sensitivity and specificity of the rabbit polyclonal CrLTP2 antibody was confirmed for CrLTP2 protein probing using immunoblotting analysis with different amounts of the purified CrLTP2 protein and the protein extract of Chlamydomonas zygote cells (Fig. S4). To prepare the samples for immunoblotting analysis, the vegetative cells and gametes of two *C. reinhardtii* strains, CC-124 (mt^−^) and CC-125 (mt^+^), and the zygotes collected at different time points were harvested with brief centrifugation and grounded into a fine powder in liquid nitrogen. The cells were lysed with 10–20 folds cell volumes of SDS sampling buffer [2% SDS (w/v), 7% glycerol, 5% 2-mercaptoethanol (v/v), and 60 mM Tris-HCl, pH 6.8] at 95 °C for 5 minutes and the whole cell proteins were resolved by 12% SDS-PAGE (w/v). The resolved proteins were transferred to polyvinylidene difluoride (PVDF) membranes in a Bjerrum Schafer-Nielsen transfer buffer (48 mM Tris, 39 mM glycine, 20% methanol, pH 9.2) by a semidry transferring cell (Bio-Rad, Hercules, CA). After blocking with TBST containing 3% nonfat milk for 1 hour [(w/v) TBST, 50 mM Tris, 150 mM NaCl, 0.1% Tween 20 (v/v), pH 7.5], the membrane was incubated with the primary antibodies (1:5000) overnight at 4 °C. Then, the membrane was incubated in a solution with HRP-conjugated antibodies for rabbit IgG (1:10,000) at room temperature for 1 hour before the signals were detected with ECL substrate kit (Pierce™/Thermo Fisher Scientific, USA).

### Protein lipid overlay assay

Sample stocks of each lipid compounds used in this assay was dissolved in a solvent mixture of methanol and chloroform (1:1, v/v) to the concentration of 5 mM. The lipid stock was diluted with a mixture of methanol, chloroform and water (2:1:0.8, v/v) to the concentration of 250 μM. Each diluted lipid sample was pre-spotted onto a PVDF membrane using glass micropipettes and the membrane was air-dried at room temperature for 30 minutes in a hood. The PVDF membrane was blocked in the blocking buffer [3% BSA (w/v) in TBST buffer] for 2 hours at room temperature, followed by being incubated in a solution of blocking buffer containing 0.6 μg/ml CrLTP2 protein overnight at 4 °C. After washing with TBST for 12 times within 1 hour at room temperature, the membrane was incubated with the anti-CrLTP2 antibodies (1:5000) and washed for another 12 times within 1 hour with TBST. Subsequently, the membrane was incubated in the TBST medium containing HPR-conjugated antibodies for rabbit IgG at a dilution of 1:5000 at room temperature for 1 hour. After the last TBST washing process for 10 times in 1 hour at room temperature, the signals were detected with ECL substrate kit.

### Protease digestion analysis

Equal amounts of the purified CrLTP2 and BSA proteins (50 μg in 100 μl of 50 mM Tris-HCl medium, pH 7.5) were mixed with 1 μg Pronase E (Sigma Aldrich, USA) and incubated at 37 °C. Small aliquots of 10 μl collected from the reacting solution at each designated time were mixed with SDS sampling buffer and resolved by 12% SDS-PAGE. The protease digestion results were visualized by staining the SDS-PAGE gel with Coomassie Brilliant Blue.

## Supplementary Information

**Additional file 1: Supplementary Figure 1.** Unrooted phylogenetic tree of the nsLTPs from land plants and green algae with sequence names. This tree is the same as the one in Figure 3 but has the sequence name of each nsLTP member on the tips. Only the bootstrap values above 50 are shown on the nodes. Each nsLTP type is indicated in a specific color: Algae nsLTPs in red, type I nsLTPs in blue, type II nsLTPs in green, type C nsLTPs in yellow, type D nsLTPs in purple, and type G nsLTPs in pink. **Supplementary Figure 2.** Transcript expression levels of *CrLTP1* and *CrLTP2* genes. The levels of CrLTPs transcripts were computed from the RNA-seq data of Chlamydomonas provided by Dr. Anthony, H. C. Huang [42]. The expression levels of CrLTP1 (black column) and CrLTP2 (grey column) in the vegetative cells, gametes, zygotes and tetra cells are shown in RPKM. **Supplementary Figure 3.** Expression and purification of CrLTP2. **A** FPLC profile of the Ni-NTA affinity purification (left panel) with the SDS-PAGE inserted are shown, as indicated: lane 1 and 2—wash with 50 mM imidazole; lane 3 and 4—wash with 100 mM imidazole; lane 5 to 6—elution with 250 mM imidazole. S: supernatant; P: pellet; F: flow-through. The Trx-CrLTP2 fusion protein is indicated with an arrow. **B** The Trx-CrLTP2 was eluted by buffer A with 250 mM imidazole followed by using TEV protease digestion to release CrLTP2 from Thioredoxin. The residual uncleaved Trx-CrLTP2 fusion protein and 6xHis-tagged TEV protease can be removed through subtractive Ni-NTA purification. Arrows indicate the uncleaved Trx-CrLTP2 (lane 1 and 2) and the pure CrLTP2 protein (lane 3 and 4). The gel pictures were taken focusing on the sample lanes as close as possible, hence, one edge of each gel in both (A) and (B) is missing in our original picture. In Figure A, right panel, the top edge is cut out of the sight, and in Figure B, the bottom edge is cut out of the sight. The images with gel edges marked are provided in Supplementary Figure 8. **Supplementary Figure 4.** Sensitivity and specificity analysis of the anti-CrLTP2 antibody. The specificity and sensitivity of the anti-CrLTP2 antibody were tested using the recombinant CrLTP2 protein and total protein extracts of Chlamydomonas zygotes. Pre-immune serum that was collected prior to the immunization of the rabbit serves as the control. Two identical data sets of the SDS-PAGE resolved protein samples containing 2.5, 5, and 10 ng purified CrLTP2 along with the protein extract of Chlamydomonas zygote cells were respectively blotted with anti-CrLTP2 serum and pre-immune serum. Arrows indicate the position of CrLTP2 protein and the numbers at left in kilo-Dolton denote the molecular mass. ng: nanogram. The blots were cut prior to hybridization with antibodies. The original PVDF membrane after immunobloting which were imaged under white light and chemiluminescent were shown in Supplementary Figure 9. The chemiluminescent images with different exposure time are shown in Supplementary Figure 10. **Supplementary Figure 5.** The RT-PCR analysis of CrLTP expression (Full-length gels of Figure 6A). The analysis of the transcript levels of *CrLTP1* and *CrLTP2* in different developmental stages of Chlamydomonas. The agarose gels were loaded with the RT-PCR products using primers sets specific to *CrLTP1, CrLTP2* and *Actin*. The arrowheads, from top to bottom, point to bands of *CrLTP1*, *CrLTP2*, and *Actin*, respectively. All the uncropped images of the gels with left, right and bottom edges and the loading wells are shown. **Supplementary Figure 6.** The immunoblotting of CrLTP2 (Full-length blots and gels with visible edges of Figure 6B). The expression pattern of CrLTP2 protein in different Chlamydomonas developmental stages, including vegetable cells, gamete cells and zygote cells, are illustrated using antibodies against CrLTP2 and α-tubulin (control). The arrowheads respectively point to the proteins bands of CrLTP2 and α-tubulin. **Supplementary Figure 7.** Proteinase digestion analysis of CrLTP2 (Full-length gel of Figure 7B). The analysis of the protease-resistant ability of CrLTP2 protein. Bovine serum albumin (BSA), the control substrates, and CrLTP2 protein were mixed, incubated with protease and collected at different time points: 0, 5, 10, 15, 30, 45, and 60 minutes. The numbers on the left indicate the protein molecular masses in kDa, and the arrows on the right indicate the positions of BSA and CrLTP2. **Supplementary Figure 8.** The full length image of Supplementary Figure 3. The protein sample of purified Trx-CrLTP2 and CrLTP2 are separated on the Tricin-SDS gel. These gel pictures were taken as close to the gel as possible to increase band sharpness, hence the margin of the picture other than the gel itself is very thin or even invisible. Both images contain the right and left edges and the protein marker in the left hand side. **Supplementary Figure 9.** Sensitivity and specificity analysis of the anti-CrLTP2 antibody (Full-length membrane of Supplementary Figure 4). The protein samples in these two blots were equally loaded for SDS-PAGE followed by being transferred to the same PVDF membrane for the same transfer efficiency. Subsequently, the PVDF membrane were cut and hybrizied with anti-CrLTP2 antibodies and preimmune serum seperatly. **Supplementary Figure 10.** The chemiluminescent images with different exposure time of Supplementary Figure 4 and Supplementary Figure 9. **Supplementary Table 1.** Gene and protein features of non-specific lipid transfer proteins found in chlorophytes and charophytes of green algae. **Supplementary Table 2.** Primers for the molecular cloning of CrLTPs genes, DNA constructs expressed in *E. coli* and RT-PCR analyses of *CrLTPs* are listed. The restriction sites are underlined, and the TEV add-ons residues are highlighted in gray.

## Data Availability

All analyzed or generated data is included in this article. The data analyzed or generated in this study can be obtained from the corresponding author with upon reasonable request. The data presented in this study are available on request from the corresponding author. All databases used in the study are open for public access, including the National Center for Biotechnology Information (NCBI, https://www.ncbi.nlm.nih.gov/) and the Joint Genome Institute (JGI, https://phytozome-next.jgi.doe.gov/). The accession numbers of the investigated genome databases for this study are listed as follows: *Chara braunii*: GCA_003427395.1; *Klebsormidium nitens*: GCA_000708835.1; *Auxenochlorella protothecoides*: GCA_000733215.1; *Chlamydomonas reinhardtii*: GCA_000002595.3; *Chlorella variabilis:* GCA_000147415.1; *Coccomyxa* sp. C-169: GCA_000258705.1; *Gonium pectoral*: GCA_001584585; *Micractinium conductrix*: GCA_002245815.2; *Monoraphidium neglectum*: GCA_000611645.1; *Ostreococcus tauri*: GCA_000214015.2; *Ostreobium quekettii*: GCA_905146915.1; *Raphidocelis subcapitata:* GCA_003203535.1; *Tetrabaena socialis*: GCA_002891735.1; *Volvox carteri*: GCA_000143455.1; *Astrephomene gubernaculifera*: GCA_021605115.1; *Bathycoccus prasinos*: GCA_002220235.1; *Chlorella variabilis* NC64A: GCA_000147415.1; *Chlorokybus atmophyticus*: GCA_009103225.1; *Dunaliella salina:* GCA_002284615.2; *Edaphochlamys debaryana*: GCA_016858145.1; *Haematococcus lacustris*: GCA_003970955.1; *Helicosporidium* sp. ATCC 50920: GCA_000690575.1; *Micromonas pusilla:* GCA_000151265.1; *Cladosiphon okamuranus*: GCA_001742925.1; *Ectocarpus siliculosus*: GCA_000310025.1; *Nemacystus decipiens* Onna-1*:* none; *Saccharina japonica:* GCA_008828725.1; *Undaria pinnatifida*: GCA_012845835.1; *Chondrus crispus:* GCA_000350225.2; *Cyanidiococcus yangmingshanensis*: GCA_013995675.1; *Cyanidioschyzon merolae*: GCA_010725195.1; *Galdieria sulphuraria*: GCF_000341285.1; *Gracilaria domingensis*: GCA_022539475.1; *Porphyridium purpureum*: GCA_008690995.1; *Porphyra umbilicalis*: GCA_002049455.2.

## Abbreviations

8CM: Eight-cysteine motif
ATIs: Amylase trypsin inhibitors
BSA: Bovine serum albumin
GPI: Glycophosphatidylinositol
HyPRP: Hybrid proline-rich proteins
nsLTPs: Non-specific lipid transfer proteins
pI: Isoelectric point
PKS1: Type I polyketide synthase
RPKM: Reads Per Kilobase per Million mapped reads
SPs: Secretory signal peptide
