# Supplementary material for: The revealing of a novel lipid transfer protein lineage in green algae
Source: BMC Plant Biol. 2023 Jan 11;23:21. doi: 10.1186/s12870-023-04040-1 (PMC9832785; doi:10.1186/s12870-023-04040-1)
Supplement: Supplementary file 1 — Additional file 1: Supplementary Figure 1. Unrooted phylogenetic tree of the nsLTPs from land plants and green algae with sequence names. This tree is the same as the one in Figure 3 but has the sequence name of each nsLTP member on the tips. Only the bootstrap values above 50 are shown on the nodes. Each nsLTP type is indicated in a specific color: Algae nsLTPs in red, type I nsLTPs in blue, type II nsLTPs in green, type C nsLTPs in yellow, type D nsLTPs in purple, and type G nsLTPs in pink. Supplementary Figure 2. Transcript expression levels of CrLTP1 and CrLTP2 genes. The levels of CrLTPs transcripts were computed from the RNA-seq data of Chlamydomonas provided by Dr. Anthony, H. C. Huang [42]. The expression levels of CrLTP1 (black column) and CrLTP2 (grey column) in the vegetative cells, gametes, zygotes and tetra cells are shown in RPKM. Supplementary Figure 3. Expression and purification of CrLTP2. A FPLC profile of the Ni-NTA affinity purification (left panel) with the SDS-PAGE inserted are shown, as indicated: lane 1 and 2—wash with 50 mM imidazole; lane 3 and 4—wash with 100 mM imidazole; lane 5 to 6—elution with 250 mM imidazole. S: supernatant; P: pellet; F: flow-through. The Trx-CrLTP2 fusion protein is indicated with an arrow. B The Trx-CrLTP2 was eluted by buffer A with 250 mM imidazole followed by using TEV protease digestion to release CrLTP2 from Thioredoxin. The residual uncleaved Trx-CrLTP2 fusion protein and 6xHis-tagged TEV protease can be removed through subtractive Ni-NTA purification. Arrows indicate the uncleaved Trx-CrLTP2 (lane 1 and 2) and the pure CrLTP2 protein (lane 3 and 4). The gel pictures were taken focusing on the sample lanes as close as possible, hence, one edge of each gel in both (A) and (B) is missing in our original picture. In Figure A, right panel, the top edge is cut out of the sight, and in Figure B, the bottom edge is cut out of the sight. The images with gel edges marked are provided in Supplementary Figure 8. Supple [file 12870_2023_4040_MOESM1_ESM.pdf]

## *Supplementary Material*

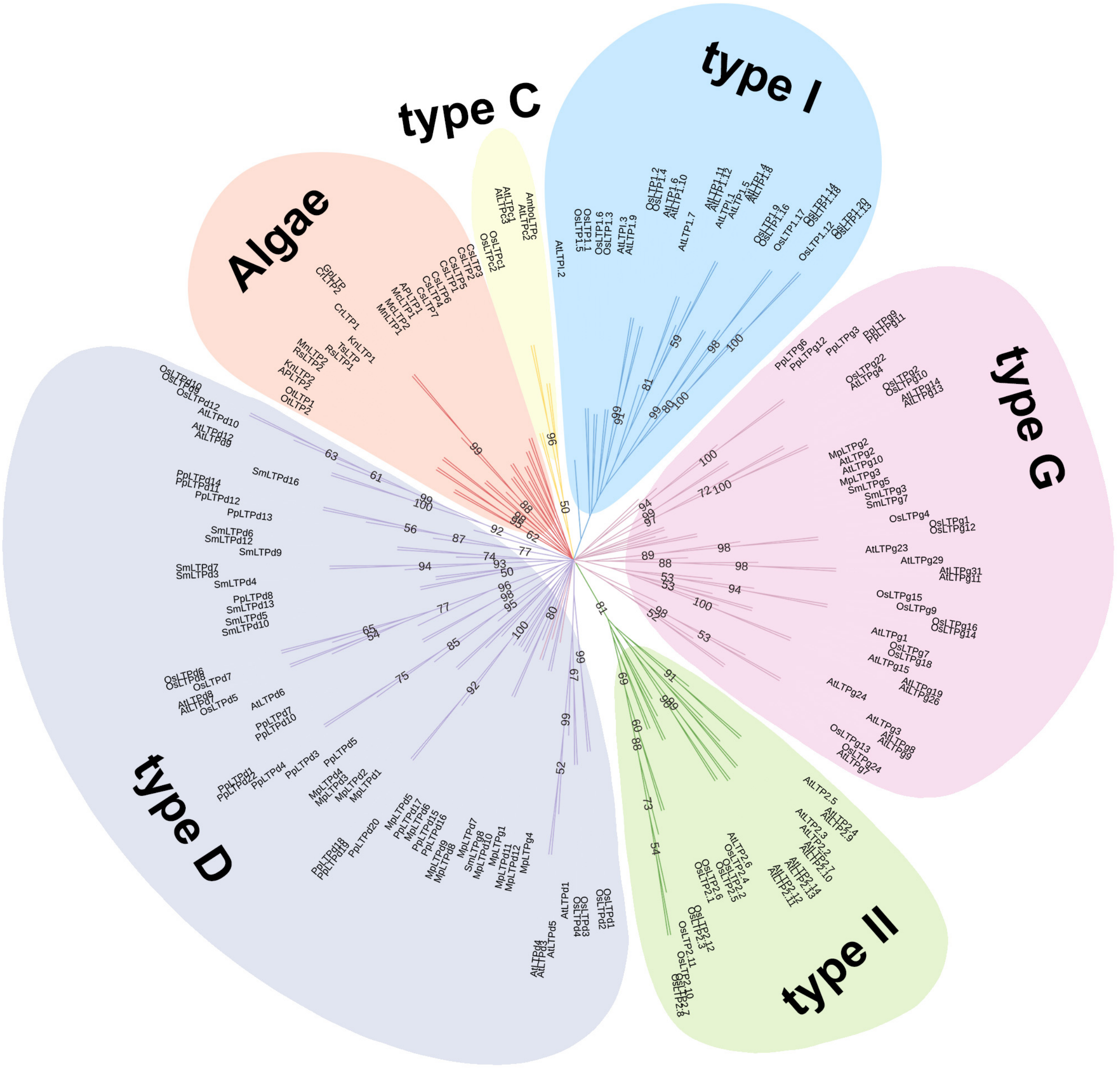


**Supplementary Figure 1. Unrooted phylogenetic tree of the nsLTPs from land plants and green algae with sequence names.** This tree is the same as the one in Figure 3 but has the sequence name of each nsLTP member on the tips. Only the bootstrap values above 50 are shown on the nodes. Each nsLTP type is indicated in a specific color: Algae nsLTPs in red, type I nsLTPs in blue, type II nsLTPs in green, type C nsLTPs in yellow, type D nsLTPs in purple, and type G nsLTPs in pink.

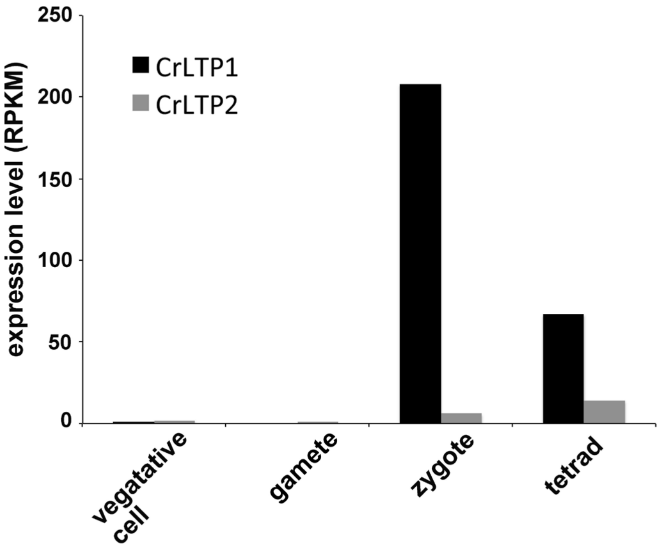


**Supplementary Figure 2**. **Transcript expression levels of *CrLTP1* and *CrLTP2* genes.** The levels of CrLTPs transcripts were computed from the RNA-seq data of Chlamydomonas provided by Dr. Anthony, H. C. Huang [42]. The expression levels of CrLTP1 (black column) and CrLTP2 (grey column) in the vegetative cells, gametes, zygotes and tetra cells are shown in RPKM.

References:

Huang, N. L., Huang, M. D., Chen, T. L. L., and Huang, A. H. C. (2013). Oleosin of subcellular lipid droplets evolved in green algae. *Plant Physiol.* 161, 1862-1874. doi: 10.1104/pp.112.212514

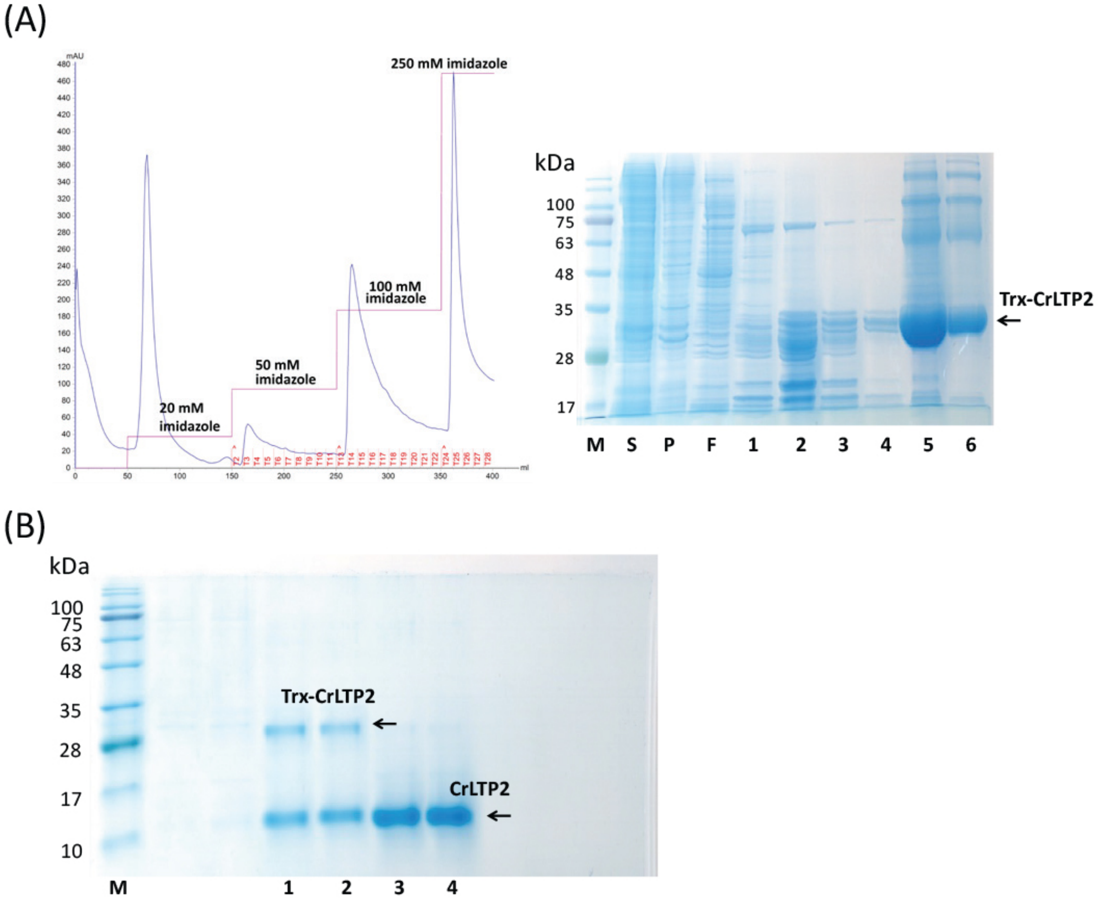


**Supplementary Figure 3. Expression and purification of CrLTP2. (A)** FPLC profile of the Ni-NTA affinity purification (left panel) with the SDS-PAGE inserted are shown, as indicated: lane 1 and 2—wash with 50 mM imidazole; lane 3 and 4—wash with 100 mM imidazole; lane 5 to 6—elution with 250 mM imidazole. S: supernatant; P: pellet; F: flow-through. The Trx-CrLTP2 fusion protein is indicated with an arrow. **(B)** The Trx-CrLTP2 was eluted by buffer A with 250 mM imidazole followed by using TEV protease digestion to release CrLTP2 from Thioredoxin. The residual uncleaved Trx-CrLTP2 fusion protein and 6xHis-tagged TEV protease can be removed through subtractive Ni-NTA purification. Arrows indicate the uncleaved Trx-CrLTP2 (lane 1 and 2) and the pure CrLTP2 protein (lane 3 and 4). The gel pictures were taken focusing on the sample lanes as close as possible, hence, one edge of each gel in both (A) and (B) is missing in our original picture. In Figure A, right panel, the top edge is cut out of the sight, and in Figure B, the bottom edge is cut out of the sight. The images with gel edges marked are provided in Supplementary Figure 8.

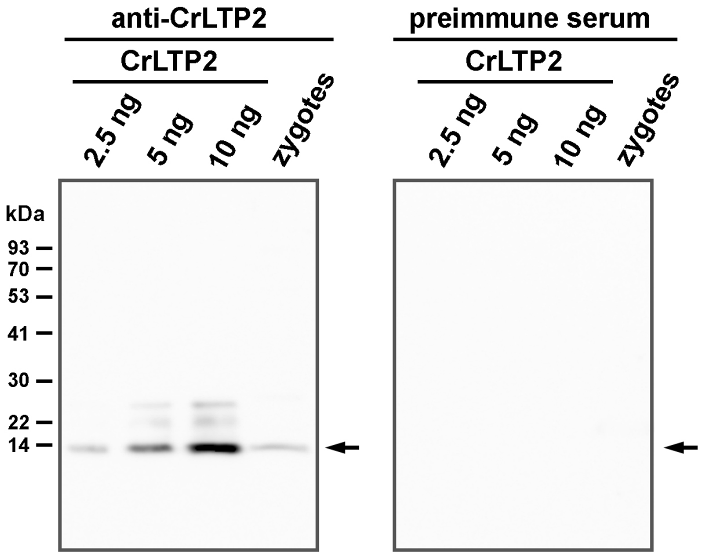


**Supplementary Figure 4. Sensitivity and specificity analysis of the anti-CrLTP2 antibody.** The specificity and sensitivity of the anti-CrLTP2 antibody were tested using the recombinant CrLTP2 protein and total protein extracts of Chlamydomonas zygotes. Pre-immune serum that was collected prior to the immunization of the rabbit serves as the control. Two identical data sets of the SDS-PAGE resolved protein samples containing 2.5, 5, and 10 ng purified CrLTP2 along with the protein extract of Chlamydomonas zygote cells were respectively blotted with anti-CrLTP2 serum and pre-immune serum. Arrows indicate the position of CrLTP2 protein and the numbers at left in kilo-Dolton denote the molecular mass. ng: nanogram. The blots were cut prior to hybridization with antibodies. The original PVDF membrane after immunobloting which were imaged under white light and chemiluminescent were shown in Supplementary Figure 9. The chemiluminescent images with different exposure time are shown in Supplementary Figure 10.

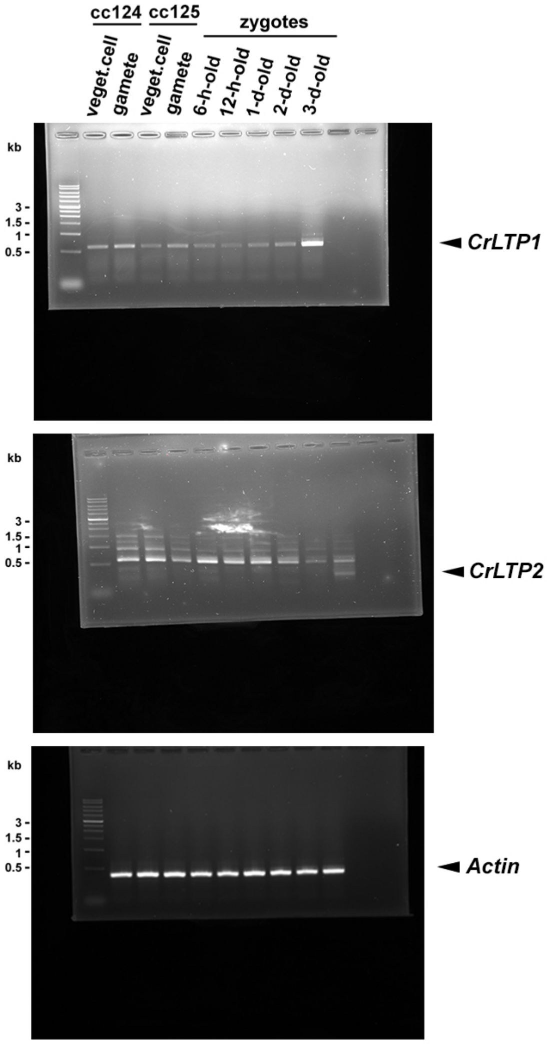


**Supplementary Figure 5. The RT-PCR analysis of CrLTP expression (Full-length gels of Figure 6A).** The analysis of the transcript levels of *CrLTP1* and *CrLTP2* in different developmental stages of Chlamydomonas. The agarose gels were loaded with the RT-PCR products using primers sets specific to *CrLTP1, CrLTP2* and *Actin*. The arrowheads, from top to bottom, point to bands of *CrLTP1*, *CrLTP2*, and *Actin*, respectively. All the uncropped images of the gels with left, right and bottom edges and the loading wells are shown.

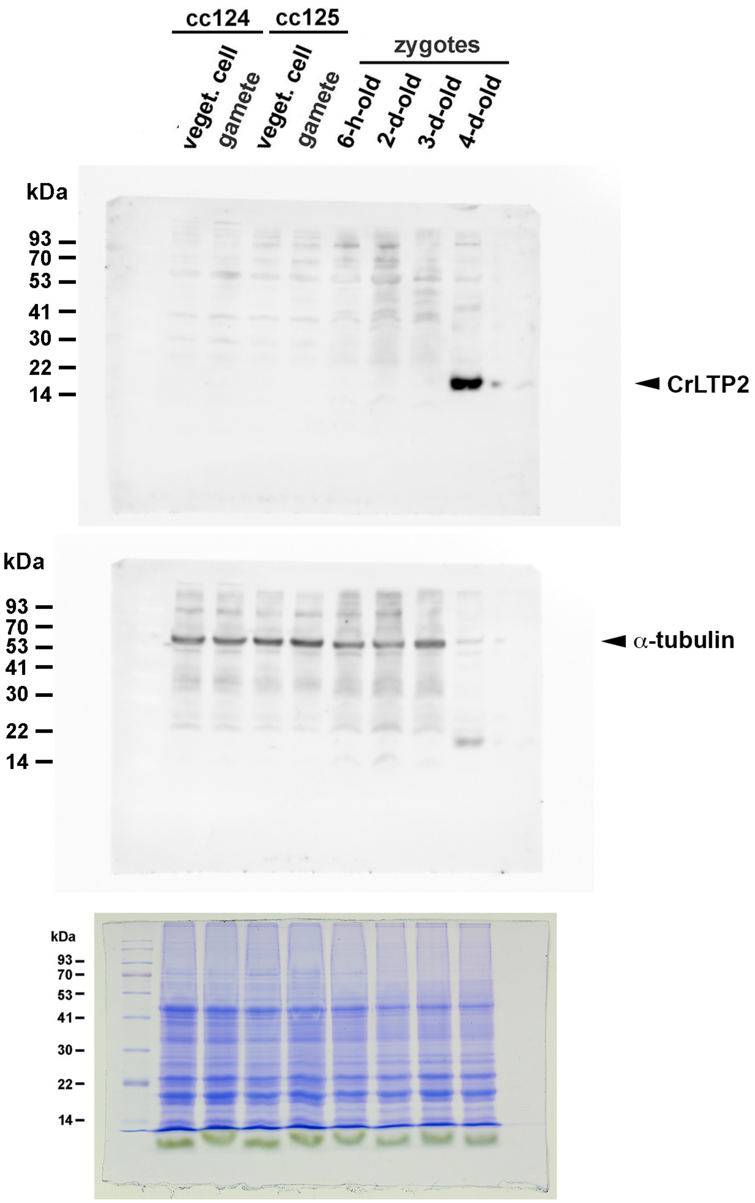


**Supplementary Figure 6. The immunoblotting of CrLTP2 (Full-length blots and gels with visible edges of Figure 6B).** The expression pattern of CrLTP2 protein in different Chlamydomonas developmental stages, including vegetable cells, gamete cells and zygote cells, are illustrated using antibodies against CrLTP2 and α-tubulin (control). The arrowheads respectively point to the proteins bands of CrLTP2 and α-tubulin.

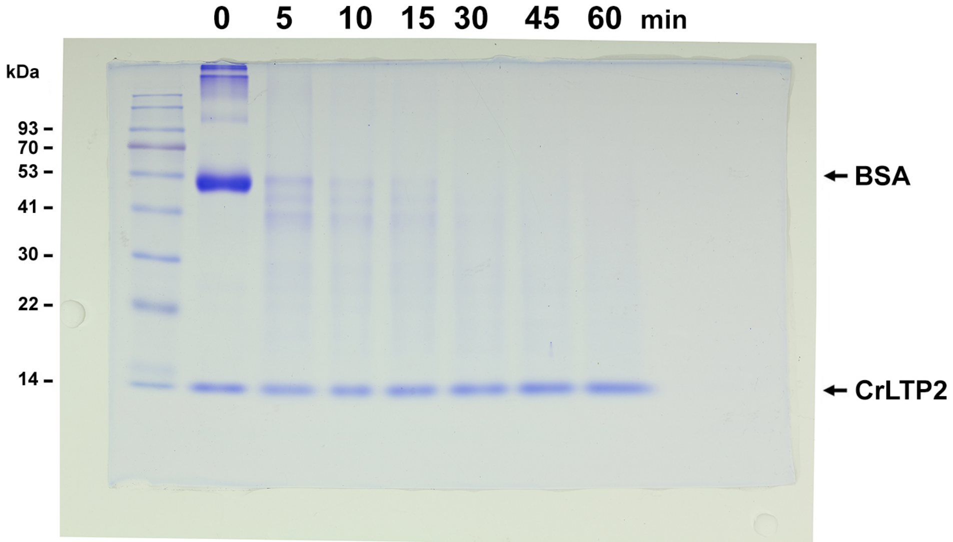


**Supplementary Figure 7. Proteinase digestion analysis of CrLTP2 (Full-length gel of Figure 7B).** The analysis of the protease-resistant ability of CrLTP2 protein. Bovine serum albumin (BSA), the control substrates, and CrLTP2 protein were mixed, incubated with protease and collected at different time points: 0, 5, 10, 15, 30, 45, and 60 minutes. The numbers on the left indicate the protein molecular masses in kDa, and the arrows on the right indicate the positions of BSA and CrLTP2.

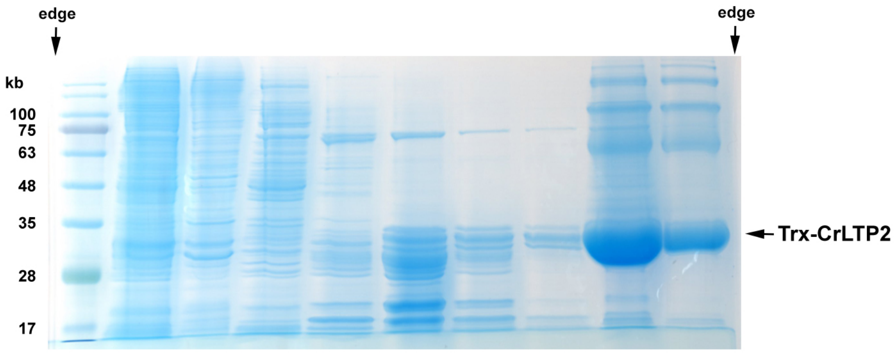


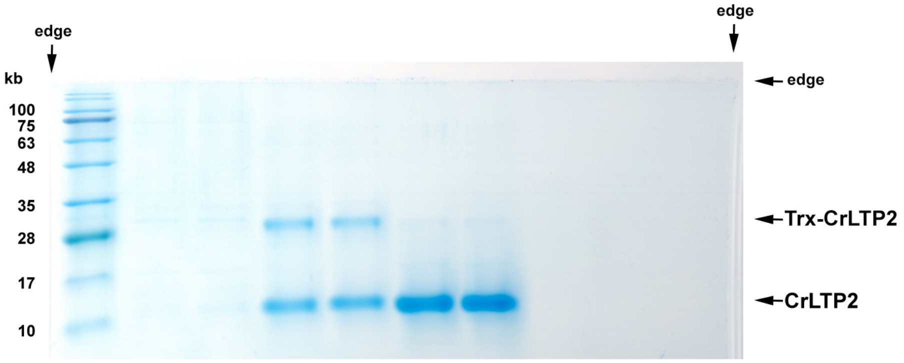


**Supplementary Figure 8. The full length image of Supplementary Figure 3. The protein sample of purified Trx-CrLTP2 and CrLTP2 are separated on the Tricin-SDS gel.** These gel pictures were taken as close to the gel as possible to increase band sharpness, hence the margin of the picture other than the gel itself is very thin or even invisible. Both images contain the right and left edges and the protein marker in the left hand side.

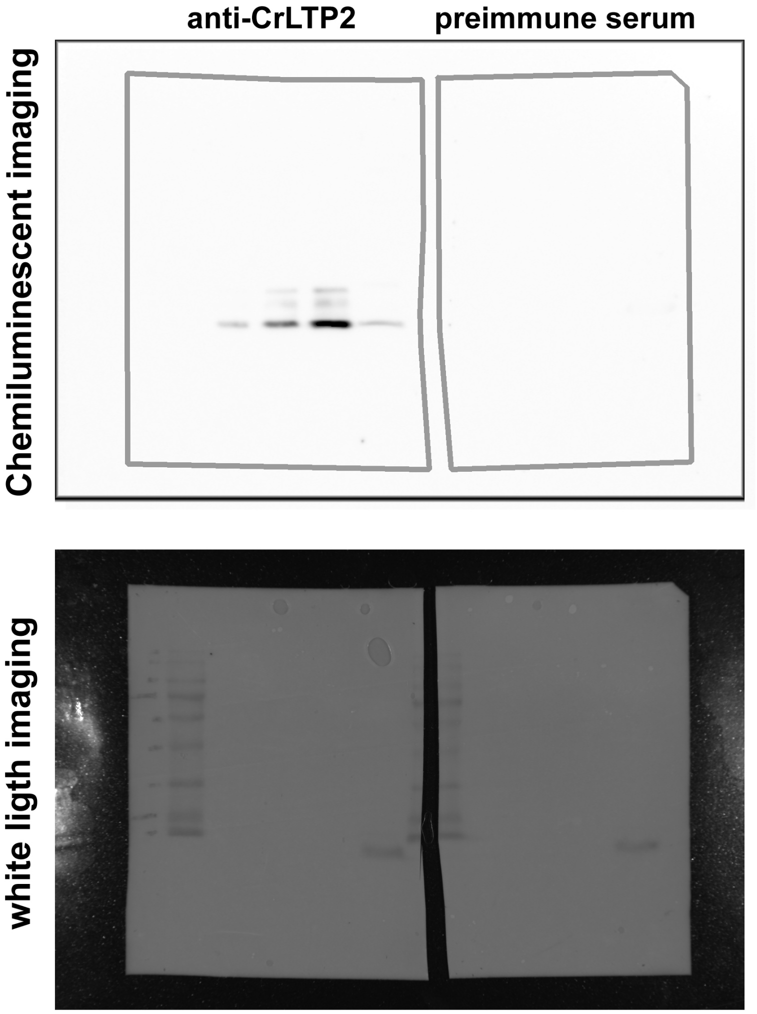


**Supplementary Figure 9. Sensitivity and specificity analysis of the anti-CrLTP2 antibody (Full-length membrane of Supplementary Figure 4).** The protein samples in these two blots were equally loaded for SDS-PAGE followed by being transferred to the same PVDF membrane for the same transfer efficiency. Subsequently, the PVDF membrane were cut and hybrizied with anti-CrLTP2 antibodies and preimmune serum seperatly.

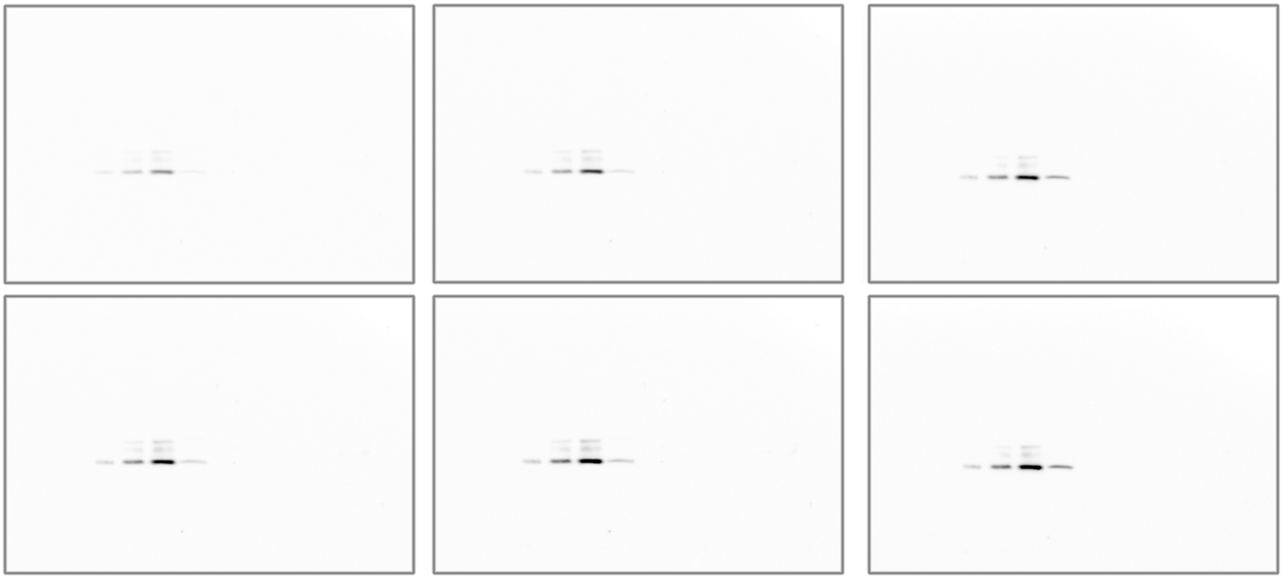

**Supplementary Figure 10. The chemiluminescent images with different exposure time of Supplementary Figure 4 and Supplementary Figure 9.**

**Supplementary Table 1.** Gene and protein features of non-specific lipid transfer proteins found in chlorophytes and charophytes of green algae.

| nsLTP genes | accession No. | No. of intron | SP | mature protein | | |
|---|---|---|---|---|---|---|
| | | | | AA | M.W. | p*I* |
| *Klebsormidium nitens* | | | | | | |
| *KnLTP1* | GAQ85394.1 | 1 | 24 | 205 | 21.02 | 7.53 |
| *KnLTP2* | GAQ82741.1 | 3 | 33 | 196 | 21.77 | 4.91 |
| *Auxenochlorella protothecoides* | | | | | | |
| *ApLTP1* | XP_011401526.1 | 2 | 20 | 108 | 11.14 | 4.52 |
| *ApLTP2* | XP_011399422.1 | 3 | 20 | 186 | 19.99 | 4.66 |
| *Chlamydomonas reinhardtii* | | | | | | |
| *CrLTP1* | PNW87352.1 | 5 | 31 | 181 | 19.9 | 5.53 |
| *CrLTP2* | PNW87351.1 | 5 | 29 | 144 | 15.19 | 8.64 |
| *Coccomyxa sp. C-169* | | | | | | |
| *CsLTP1* | XP_005643308.1 | 4 | 19 | 122 | 12.58 | 6.15 |
| *CsLTP2* | XP_005643303.1 | 5 | 24 | 127 | 13.39 | 5.11 |
| *CsLTP3* | XP_005651742.1 | 3 | 25 | 104 | 10.78 | 7.63 |
| *CsLTP4* | XP_005644114.1 | 4 | 23 | 116 | 11.91 | 6.74 |
| *CsLTP5* | XP_005643309.1 | 4 | 19 | 126 | 13.32 | 6.27 |
| *CsLTP6* | XP_005644407.1 | 4 | 21 | 126 | 12.99 | 7.73 |
| *CsLTP7* | XP_005647951.1 | 4 | 25 | 117 | 11.64 | 5.04 |
| *CsLTP8* | XP_005648896.1 | 8 | 21 | 412 | 44.16 | 4.61 |
| *Chlorella variabilis* | | | | | | |
| *CvLTP1* | XP_005852285.1 | 3 | 30 | 163 | 16.54 | 4.11 |
| *CvLTP2* | XP_005851679.1 | 5 | 20 | 112 | 13.46 | 5.08 |
| *Gonium pectoral* | | | | | | |
| *GpLTP1* | KXZ54101.1 | 4 | 30 | 151 | 15.83 | 8.18 |
| *Micractinium conductrix* | | | | | | |
| *McLTP1* | PSC75666.1 | 5 | 25 | 135 | 14.47 | 4.80 |
| *McLTP2* | PSC68779.1 | 3 | 26 | 116 | 11.19 | 4.27 |
| *Monoraphidium neglectum* | | | | | | |
| *MnLTP1* | XP_013906595.1 | 4 | 23 | 100 | 10.54 | 9.18 |
| *MnLTP2* | XP_013895246.1 | 3 | 25 | 484 | 49.69 | 8.66 |
| *Ostreobium quekettii* | | | | | | |
| *OqLTP1* | CAD7704259.1 | 4 | 20 | 223 | 22.58 | 4.32 |
| *OqLTP2* | CAD7705092.1 | 3 | 20 | 197 | 20.18 | 4.24 |
| *Ostreococcus tauri* | | | | | | |
| *OtLTP1* | OUS48719.1 | 0 | 19 | 394 | 42.03 | 4.89 |
| *OtLTP2* | XP_022839860.1 | 0 | 23 | 459 | 50.28 | 5.52 |
| *Raphidocelis subcapitata* | | | | | | |
| *RsLTP1* | GBF90531.1 | 2 | 21 | 174 | 16.14 | 7.72 |
| *RsLTP2* | GBF97693.1 | 3 | 22 | 101 | 10.36 | 7.75 |
| *Tetrabaena socialis* | | | | | | |
| *TsLTP* | PNH05028.1 | 6 | 29 | 340 | 33.08 | 5.72 |
| *Volvox carteri* | | | | | | |
| *VcLTP* | XP_002952036.1 | 3 | 28 | 161 | 15.99 | 7.60 |

**Supplementary Table 2.** Primers for the molecular cloning of CrLTPs genes, DNA constructs expressed in *E. coli* and RT-PCR analyses of *CrLTPs* are listed. The restriction sites are underlined, and the TEV add-ons residues are highlighted in gray.

| Primer name | Sequence (5' → 3') |
|---|---|
| **Cloning of CrLTP1 by nested RT-PCR** | |
| CrLTP1-F | CCGCCTCGACAACCTGAC |
| CrLTP1-R | CATCGCCGATACCAACCTATC |
| CrLTP1-nested-F | ATGGCGCCCCCACCGC |
| CrLTP1-nested-R | CTAGATTGGGTGCTCCGGGC |
| **Cloning of CrLTP2 by nested RT-PCR** | |
| CrLTP2-F | ACCTTCTATCCGAGCAAGCAAG |
| CrLTP2-R | GCCCAAGCAATCTATCTATGGC |
| CrLTP2-nested-F | ATGGCCGTCCACTTCAGCAC |
| CrLTP2-nested-R | CAATCTATCTATGGCTTCTTCTTGCC |
| **Generation of the construct for CrLTP2 expression in *E. coli*** | |
| CrLTP2-KpnI-TEV-F | CTGGGTACCGAGAACCTGTACTTCCAATCCAATATTGAGTGCATCCAGGCGGGG |
| CrLTP2-XhoI-R | GTGCTCGAGCTATGGCTTCTTCTTGCCGGC |
| **RT-PCR analysis of CrLTPs** | |
| CrLTP1-RT-F | CGCTATCTCTGGTGCTTCTGG |
| CrLTP1-RT-R | TCCATGTAAACCACGGGAATC |
| CrLTP2-RT-F | GCAGTTGTGGTCGTCGTGG |
| CrLTP2- RT-R | CAATCTATCTATGGCTTCTTCTTGCC |
